# The NF-Y Transcription Factor Family in Watermelon: Re-Characterization, Assembly of ClNF-Y Complexes, Hormone- and Pathogen-Inducible Expression and Putative Functions in Disease Resistance

**DOI:** 10.3390/ijms232415778

**Published:** 2022-12-12

**Authors:** Siyu Jiang, Hui Wang, Ya Wen, Jiayu Liang, Dayong Li, Fengming Song

**Affiliations:** Key Laboratory of Crop Diseases and Insect Pests of Ministry of Agriculture, Institute of Biotechnology, Zhejiang University, Hangzhou 310058, China

**Keywords:** watermelon (*Citrullus lanatus* L.), nuclear factor Y (NF-Y), ClNF-Y complex, expression pattern, disease resistance

## Abstract

Nuclear factor Y (NF-Y) is a heterotrimeric transcription factor that binds to the CCAAT *cis*-element in the promoters of target genes and plays critical roles in plant growth, development, and stress responses. In the present study, we aimed to re-characterize the *ClNF-Y* family in watermelon, examine the assembly of ClNF-Y complexes, and explore their possible involvement in disease resistance. A total of 25 *ClNF-Y* genes (7 *ClNF-YAs*, 10 *ClNF-YBs*, and 8 *ClNF-YCs*) were identified in the watermelon genome. The *ClNF-Y* family was comprehensively characterized in terms of gene and protein structures, phylogenetic relationships, and evolution events. Different types of *cis*-elements responsible for plant growth and development, phytohormones, and/or stress responses were identified in the promoters of the *ClNF-Y* genes. ClNF-YAs and ClNF-YCs were mainly localized in the nucleus, while most of the ClNF-YBs were localized in the cytoplasm of cells. ClNF-YB5, -YB6, -YB7, -YB8, -YB9, and -YB10 interacted with ClNF-YC2, -YC3, -YC4, -YC5, -YC6, -YC7, and -YC8, while ClNF-YB1 and -YB3 interacted with ClNF-YC1. A total of 37 putative ClNF-Y complexes were identified, e.g., ClNF-YA1, -YA2, -YA3, and -YA7 assembled into 13, 8, 8, and 8 ClNF-Y complexes with different ClNF-YB/-YC heterodimers. Most of the *ClNF-Y* genes responded with distinct expression patterns to defense hormones such as salicylic acid, methyl jasmonate, abscisic acid, and ethylene precursor 1-aminocyclopropane-1-carboxylate, and to infection by the vascular infecting fungus *Fusarium oxysporum* f. sp. *niveum*. Overexpression of *ClNF-YB1*, *-YB8*, *-YB9*, *ClNF-YC2*, and *-YC7* in transgenic Arabidopsis resulted in an earlier flowering phenotype. Overexpression of *ClNF-YB8* in Arabidopsis led to enhanced resistance while overexpression of *ClNF-YA2* and *-YC2* resulted in decreased resistance against *Botrytis cinerea*. Similarly, overexpression of *ClNF-YA3*, *-YB1*, and *-YC4* strengthened resistance while overexpression of *ClNF-YA2* and *-YB8* attenuated resistance against *Pseudomonas syringae* pv. *tomato* DC3000. The re-characterization of the *ClNF-Y* family provides a basis from which to investigate the biological functions of *ClNF-Y* genes in respect of growth, development, and stress response in watermelon, and the identification of the functions of some *ClNF-Y* genes in disease resistance enables further exploration of the molecular mechanism of *ClNF-Ys* in the regulation of watermelon immunity against diverse pathogens.

## 1. Introduction

Upon perception of internal and external cues, plants often initiate a complicated and fine-tuned transcriptional reprogramming network to modify the expression of specific sets of genes that are involved in growth, development, and stress response [1,2]. This transcriptional reprogramming of gene expression in plants requires the concerted action of epigenetic mechanisms (e.g., DNA methylation and histone modifications) and cooperative functions of diverse transcription factors (TFs) belonging to different families in both temporal and spatial manners [2,3,4]. Among the TF families, Nuclear Factor Y (NF-Y) TFs, also known as heme activator proteins (HAPs) or CCAAT-binding factors (CBFs), play critical regulatory roles in almost all aspects of plant growth, development, and stress response through transcriptionally modulating the expression of different sets of genes via binding to the CCAAT *cis*-element in the promoters of target genes [5,6,7].

NF-Y TFs are heterotrimeric complexes and constitute an evolutionarily conserved ubiquitous family present in nearly all eukaryotes [6]. The heterotrimeric NF-Y complex consists of three subunits: NF-YA (CBF-B/HAP2), NF-YB (CBF-A/HAP3), and NF-YC (CBF-C/HAP5) [8]. Whereas each of the NF-Y subunit is encoded by a single gene in mammals and yeasts [9], they are encoded by relatively small gene families in plants [7]. For example, there are 36 *AtNF-Y* genes in Arabidopsis (10 *AtNF-YAs*, 13 *AtNF-YBs*, and 13 *AtNF-YCs*) [10] and 34 *OsNF-Y* genes in rice (11 *OsNF-YAs*, 11 *OsNF-YBs*, and 12 *OsNF-YCs*) [11]. NF-Y families have also been characterized in cucumber, soybean, maize, barley, tomato, potato, oilseed rape, and other plant species at the genome-wide level [12,13,14,15,16,17,18]. The NF-YA and NF-YC subunits have nuclear localization signals (NLSs) and are targeted to the nucleus; by contrast, the NF-YB subunits normally lack NLSs and are thus not localized in the nucleus [19]. Generally, the NF-YA subunits are localized in nucleus, where they bind to the CCAAT *cis*-elements in the promoters of their target genes [7,20,21]. By contrast, NF-YB and NF-YC subunits do not harbor the DNA-binding domains but contain the conserved histone fold motif (HFM) or histone fold domain (HFD). The HFM domains, which are formed by three α-helices, contribute to the DNA binding ability and the formation of the NF-Y complexes via protein–protein interactions [7,22,23,24]. The NF-YB and NF-YC subunits form heterodimers in the cytoplasm [22], and then translocate into the nucleus, where they interact with NF-YA subunits to complete the assembly of the heterotrimeric NF-Y complex [25,26].

The CCAAT *cis*-element is estimated to be present in ~30% of the promoters in eukaryotic genes [27]. It is thus likely that the NF-Y family plays diverse but critical roles in a wide range of biological processes in plants, including growth, development, and stress response [6,28]. Functional studies have shown that NF-Ys play key roles in the regulation of seed development and germination [29,30], root development and architecture [31,32], flowering time [33,34,35,36,37,38,39], and fruit ripening [16,40]. NF-Ys are widely involved in drought and salt stress response, via abscisic acid (ABA)-dependent or -independent pathways [6,28]; for example, modification of a single *NF-Y* gene through either overexpression or knockout significantly improved drought tolerance in Arabidopsis, rice, maize, soybean, and *Populus* [41,42,43,44,45,46,47]. However, the involvement of NF-Ys in both beneficial and harmful plant–microbe interactions has also been documented. In leguminous plants, *Medicago truncatula* MtNF-YA1 and MtNF-YA2; *Phaseolus vulgaris* PvNF-YA1, PvNF-YB7, and PvNF-YC1; and *Lotus japonicus* LjNF-YA1 and LjNF-YB1 are the key regulators of symbiotic root nodule development [48,49,50,51,52,53]. In nonlegume *Parasponia andersonii*, PaNF-YA1 acts as a core genetic network in nitrogen-fixing nodule symbioses [54]. Several lines of evidence have suggested the implication of *NF-Ys* in plant immunity against different pathogens. Overexpression of rice *OsNF-YA2* (*OsHAP2E*) conferred enhanced resistance to *Magnaporthe oryzae* and *Xanthomonas oryzae* infections [44], while overexpression of *OsNF-YA1*, *-YA2*, or *-YA10* increased susceptibility to infections by two viruses, rice stripe virus and southern rice black-streaked dwarf virus, through suppressing jasmonic acid (JA)-mediated signaling [55]. Soybean plants with overexpression of *GmNF-YC4-2* increased broad disease resistance to bacterial, viral, and fungal infections by *bean pod mottle virus*, *Pseudomonas syringae* pv. *glycinea*, and *Fusarium virguliforme* [56]. *Medicago truncatula* MtNF-YA1, a key determinant for nodule development and establishment of rhizobial symbiosis, was found to be a negative regulator of resistance against *Aphanomyces euteiches*, a root pathogenic oomycete [57]. The cassava MeNF-Y complexes MeNF-YA1/3, MeNF-YB11/16, and MeNF-YC11/12 were shown to play roles in transcriptional activation of the defense response against a bacterial pathogen *Xanthomonas axonopodis* pv. *manihotis* [58].

Watermelon (*Citrullus lanatus* L.), providing popular fresh fruit, is an important horticultural crop worldwide. Fusarium wilt, caused by the soil-borne fungus *Fusarium oxysporum* f. sp. *niveum* (*Fon*), is one of the most devasting diseases that leads to great annual yield losses [59]; however, little is known about the genetic and molecular mechanisms governing the resistance against Fusarium wilt in watermelon. Previously, 19 *ClNF-Y* genes were identified and the expression of 13 *ClNF-Y* genes was found to be affected by drought and salt stress [60].

In the present study, we aimed to re-characterize the watermelon *ClNF-Y* family, analyze subcellular localization, assembly of ClNF-Y complexes, and expression changes in response to hormones and *Fon*, and explore the possible involvement of the *ClNF-Y* family in disease resistance. A total of 37 putative ClNF-Y complexes were identified. The expression levels of the *ClNF-Y* genes were changed after treatment with salicylic acid (SA), methyl jasmonate (MeJA), ABA, and ethylene precursor 1-aminocyclopropane-1-carboxylate (ACC), or infection by *Fon*. Functional analyses through ectopic overexpression in Arabidopsis revealed that *ClNF-YA2*, *-YA3*, *-YB1*, *-YB8*, *-YC2*, and *-YC4* play roles in disease resistance. The re-characterization of the *ClNF-Y* family, the definition of putative ClNF-Y complexes, and the identification of the functions of some *ClNF-Y* genes in disease resistance provide a basis from which to further investigate the biological functions and molecular mechanisms of the *ClNF-Y* genes in growth, development, and disease resistance against diverse pathogens in watermelon.

## 2. Results

### 2.1. Identification of the Watermelon ClNF-Y Family

Nineteen *ClNF-Y* genes have been identified previously [60]. To further identify the watermelon *ClNF-Y* genes, the well-characterized Arabidopsis AtNF-Y and rice OsNF-Y protein sequences [10,11] were used as queries to search against the watermelon genome database at CuGenDB [61,62]. As a result, 7, 10, and 8 non-redundant sequences were identified to encode for putative ClNF-YA, -YB, and -YC subunits in the watermelon genome, and for convenience, unique identities were assigned as *ClNF-YA1-7*, *ClNF-YB1-10*, and *ClNF-YC1-8*, respectively, according to their chromosomal locations (Table 1). Sequence analysis revealed that the ORF lengths of *ClNF-YAs*, *ClNF-YBs*, and *ClNF-YCs* range from 666 bp (*ClNF-YA1*) to 1230 bp (*ClNF-YA2*), 405 bp (*ClNF-YB4*) to 678 bp (*ClNF-YB6*), and 354 bp (*ClNF-YC5*) to 852 bp (*ClNF-YC8*), and accordingly, the sizes of the deduced proteins vary from 221 amino acids (aa) to 409 aa, 134 aa to 225 aa, and 117 aa to 283 aa, with molecular weights of 24.4–44.3 kDa, 15.5–24.7 kDa, and 13.0–31.7 kDa and isoelectric points (*p*I) of 6.49–9.14, 4.57–7.98, and 4.77–9.17, respectively (Table 1).

**Figure 1:**
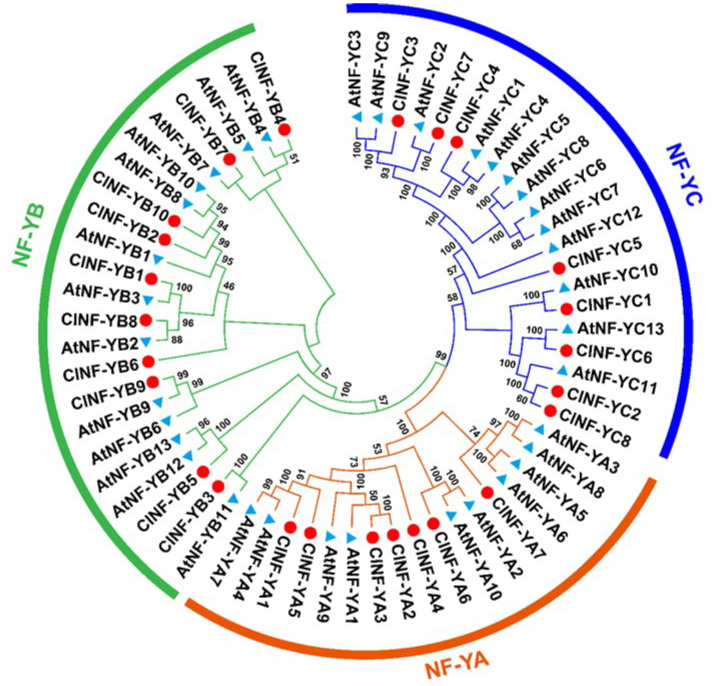
Phylogenetic relationships of the watermelon ClNF-Y proteins with Arabidopsis AtNF-Y proteins. The phylogenetic tree was constructed via Clustal X1.8 and MEGA 7.0 software using the neighbor-joining method with 1000 bootstrap replicates. The red circles and blue triangles represent the ClNF-Y and AtNF-Y proteins, respectively.

Phylogenetic tree analysis with the well-characterized Arabidopsis AtNF-Y proteins revealed that the 25 ClNF-Y proteins were distinctly classified into three major groups: NF-YA, NF-YB, and NF-YC (Figure 1). In the phylogenetic tree, most of the ClNF-Y proteins are clustered with 1 or 2 AtNF-Y proteins, except for ClNF-YA7, ClNF-YB6, and ClNF-YC5, which are not closely clustered with AtNF-Ys (Figure 1).

### 2.2. Structural Features of ClNF-Y Genes and Proteins

**Figure 2:**
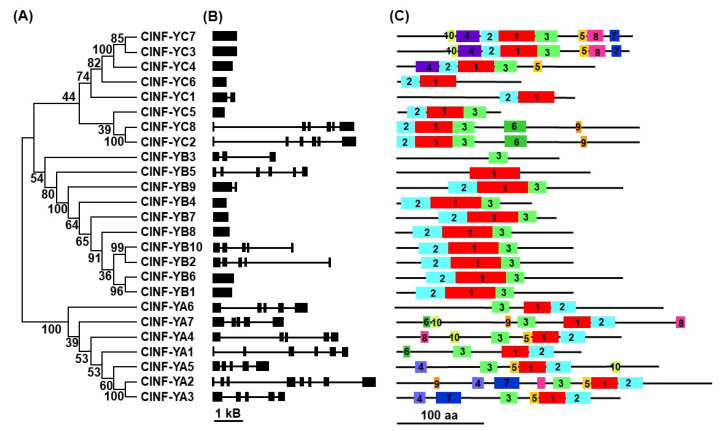
Phylogenetic relationships, gene structure, and conserved motifs in ClNF-Y proteins. (**A**) Phylogenetic relationships between the 25 ClNF-Y proteins. (**B**) Structures of the watermelon *ClNF-Y* genes. Exons and introns are indicated by filled boxes and thin lines, respectively. (**C**) Organization of conserved motifs in ClNF-Y proteins. Different colored boxes with numbers represent different motifs (see Figure S2 for details).

The 25 *ClNF-Y* genes are unevenly distributed on ten chromosomes in the watermelon genome and chromosome 5 does not harbor any *ClNF-Y* gene (Table 1; Figure S1). Notably, chromosomes 2 and 10 host 6 (1 for *ClNF-YA2*; 3 for *ClNF-YB1*, *-YB2*, and *-YB3*; and 2 for *ClNF-YC1* and *-YC2*) and 4 (1 for *ClNF-YA6*, and 3 for *ClNF-YB8*, *-YB9*, and *-YB10*) *ClNF-Y* genes, respectively (Table 1; Figure S1). The structure of the *ClNF-Y* genes in the CDS is highly divergent in terms of the exon and intron numbers. Ten *ClNF-Y* genes (5 for *ClNF-YB1*, *-YB4*, *-YB6*, *-YB7*, and *-YB8*; and 5 for *ClNF-YC3*, *-YC4*, *-YC5, -YC6*, and *-YC7*) are intronless, *ClNF-YB9* and *ClNF-YC1* contain a single intron, and the remaining genes harbor 2 (*ClNF-YB3*) to 7 (*ClNF-YA2*) introns (Figure 2B). Among the three subfamilies, the *ClNF-YA* genes tend to have more complicated exon–intron organization with at least 4 introns (Figure 2A,B).

MEME analysis revealed that ClNF-YAs, ClNF-YBs, and ClNF-YCs contain 10, 3, and 10 putative conserved motifs, respectively (Figure 2C; Figure S2). The ClNF-YA proteins contain motifs 1 and 2, representing the NF-YB/YC interaction and DNA-binding domains, respectively, which constitute conserved regions composed of approximately 70 aa (Figure S3A). ClNF-YB1, -YB2, -YB4, -YB6, -YB7, -YB8, -YB9, and -YB10 contain motifs 1, 2, and 3, representing the NF-YA interaction, DNA binding, and NF-YC interaction domains, respectively, which constitute the highly conserved HFM domain composed of approximately 92 aa (Figure S3B). However, ClNF-YB5 only contains motif 1 but lacks motifs 2 and 3, while ClNF-YB3 only contains motif 3 but lacks motifs 1 and 2, implying that both of them may be incomplete proteins or differ from other ClNF-YBs in terms of their biochemical activity. ClNF-YC2, -YC3, -YC4, -YC5, -YC6, -YC7, and -YC8 contain motifs 1, 2, and 3, representing the NF-YA interaction (also DNA-binding domain), NF-YB interaction, and NF-YA interaction domains, respectively, which constitute the highly conserved HFM domain composed of approximately 83 aa (Figure S3C). Notably, ClNF-YC1 only contains motifs 1 and 2 but lacks motif 3 (Figure S3C). Furthermore, the HFD domains in ClNF-YBs and ClNF-YC are formed by a minimum of three α-helices (α1, α2, and α3) (Figure S3B,C). Other conserved motifs were also identified in ClNF-YAs and ClNF-YCs (Figure 2C), implying the diversity and complexity of the biochemical mechanisms of the ClNF-Ys in watermelon.

### 2.3. Evolution of the ClNF-Y Family

**Figure 3:**
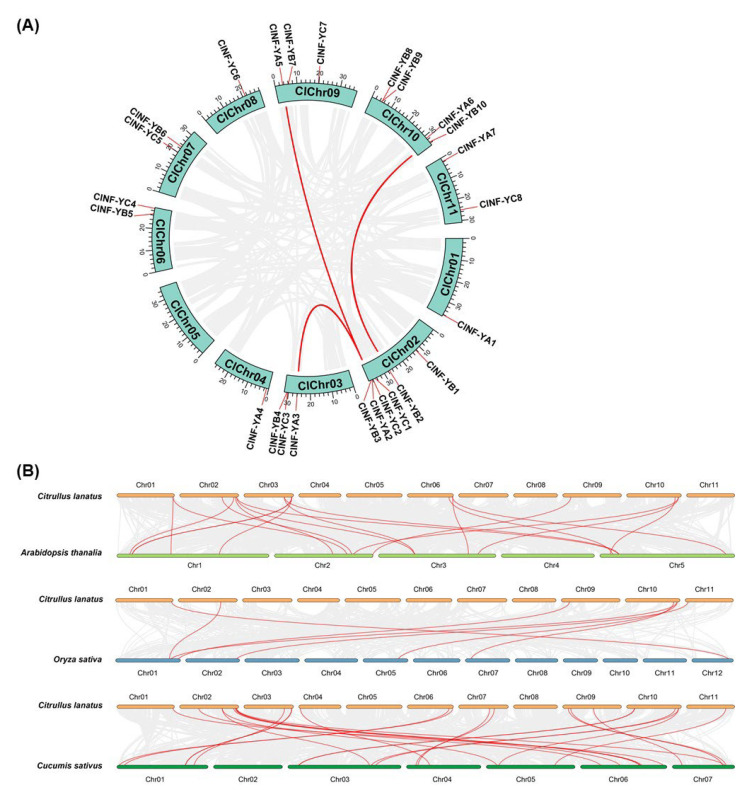
Interchromosomal and syntenic relationships between watermelon *ClNF-Y* genes. (**A**) Interchromosomal relationships between the *ClNF-Y* genes. Gray lines indicate the syntenic blocks in the genome, and red lines indicate the duplication of the *ClNF-Y* gene pairs. (**B**) Syntenic relationships between the *ClNF-Y* genes and *NF-Y* genes in other plant species. Gray lines in the background indicate the collinear blocks within the watermelon and other plant genomes, while red lines highlight the syntenic *NF-Y* gene pairs between watermelon and other plant species.

To gain insights into the expansion of the *ClNF-Y* family, the syntenic relationships between the *ClNF-Y* genes in the watermelon genome were examined. The results show that no tandem duplication event was detected but segmental duplication events for three gene pairs, *ClNF-YA2*/*-YA3*, *ClNF-YA2*/*-YA5*, and *ClNF-YB2*/*-YB10*, were identified in the *ClNF-Y* family (Figure 3A), implying that the segmental duplication was the major force that drove the expansion of the *CLNF-Y* family. The nonsynonymous (Ka)/synonymous (Ks) ratios (Ka/Ks) of gene pairs *ClNF-YA2*/-*YA3*, *ClNF-YA2*/-*YA5*, and *ClNF-YB2*/*-YB10* were estimated to be 0.2315, 0.3257, and 0.1122 (Table S1), respectively, indicating that these gene pairs evolved through purifying selection in watermelon.

Interspecific comparative syntenic maps between the watermelon *ClNF-Y* genes and the *NF-Y* genes from Arabidopsis, rice, and cucumber were constructed to further elucidate the expansion mechanism of the *ClNF-Y* family. Interspecific collinearity analyses revealed that there were strong orthologs in the *NF-Y* families among watermelon, Arabidopsis, cucumber, and rice, and identified 19, 27, and 7 collinear gene pairs between watermelon and Arabidopsis, cucumber, and rice (Figure 3B; Table S2). There are 23, 13, and 6 *ClNF-Y* genes with collinear orthologous gene pairs in cucumber, Arabidopsis, and rice genomes, respectively (Figure 3B; Table S2). Notably, five *ClNF-Y* genes, *ClNF-YA1*, *-YA6*, *-YB2*, *-YB7*, and *-YB10*, have the same collinear orthologous gene pairs in cucumber, Arabidopsis, and rice genomes (Figure 3B; Table S2), indicating that these five *ClNF-Y* genes may originate from common ancestors and were preserved during the evolution of plant species. With the exception of *ClNF-YA7* and *-YC6*, the majority of the *ClNF-Y* genes have collinear orthologous gene pairs in the cucumber genome (Figure 3B; Table S2), indicating that the *NF-Y* family is highly homologous among the cucurbit plants. The Ka/Ks ratios of the *ClNF-Y* collinear gene pairs identified in watermelon with those in rice, Arabidopsis, and cucumber are less than one (Table S2), suggesting that the watermelon *ClNF-Y* genes may have suffered strong purifying selective pressure during evolution.

### 2.4. Cis-Elements in Promoters of the ClNF-Y Genes

**Figure 4:**
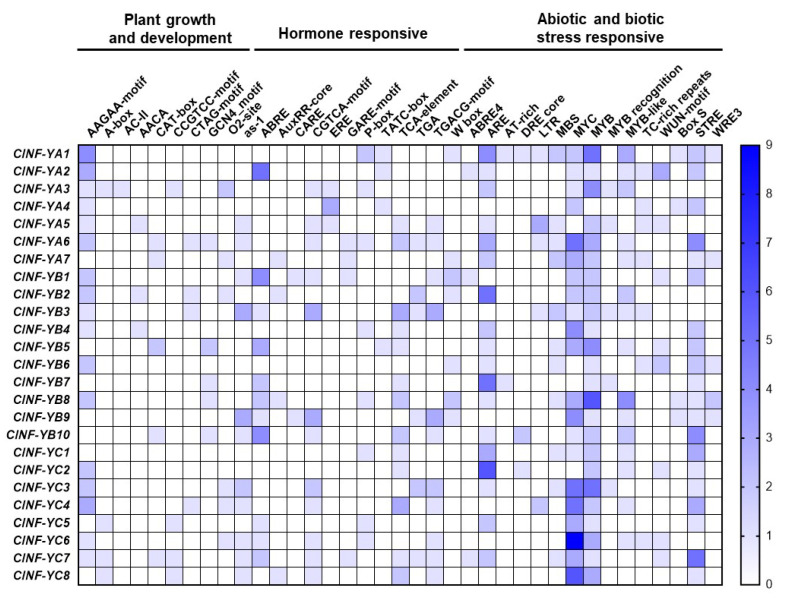
*Cis*-elements present in promoters of the watermelon *ClNF-Y* genes. Different colors indicate the numbers of the *cis*-elements in promoters of the *ClNF-Y* genes, as shown in the right color scale. See Table S3 for detail.

To gain insights into the responsiveness of the *ClNF-Y* genes, putative *cis*-elements in 1.5 Kb promoter regions of each *ClNF-Y* gene were analyzed using PlantCARE [63]. More than 2600 *cis*-elements belonging to 89 types were identified in promoters of the *ClNF-Y* genes, and apart from the common CAAT-box and TATA-box *cis*-elements, each of the *ClNF-Y* gene promoters contain an average of ~45 *cis*-elements (Table S3). A large number of light-responsive *cis*-elements such as Box 4, G-box, GATA-motif, and GT1-motif are present in almost all promoters of the *ClNF-Y* genes (Figure S4). Importantly, different types of *cis*-elements responsible for plant growth and development and phytohormones, as well as abiotic and biotic stresses, were identified in the promoters of all of the *ClNF-Y* genes (Figure 4). The *cis*-elements involved in plant growth and development included the development-related motif AAGAA, the senescence-related A-box, the vascular-specific element AC-II, the meristem expression motifs CAT-box and CCGTCC, the endosperm expression motif GCN4, and the zein metabolism regulation motif O2-site (Figure 4). The *cis*-elements involved in plant hormone response include the ABA-responsive element ABRE; the auxin-responsive elements AuxRR-core and TGA-element; gibberellin-responsive motifs CARE, GARE, P-box, and TATC; the ethylene-responsive element ERE; the SA-responsive elements TCA, SARE, and W box; and the MeJA-responsive motifs CGTCA and TGACG (Figure 4). The *cis*-elements involved in abiotic and biotic stress response included ABRE4, ARE (anaerobic induction), AT-rich (defense activation), DRE core (dehydration-responsive), LTR (low-temperature responsive), MBS, MYC, MYB, MYB recognition, MYB-like, TC-rich (defense and stress responsive), WUN-motif (wound-responsive), box S (pathogen-inducible), STRE, and WRE3 (Figure 4). In particular, 13 promoters of the *ClNF-Y* genes contain the SA-responsive TCA elements, 12 promoters harbor the MeJA-responsive element CGTCA-motif, 14 promoters carry the ethylene-responsive element ERE, and 11 promoters possess the ABA-responsive element ABRE (Figure 4). These data indicate the involvement of the *ClNF-Y* genes in plant growth, development, and stress response.

### 2.5. Subcellular Localization of the ClNF-Y Proteins

**Figure 5:**
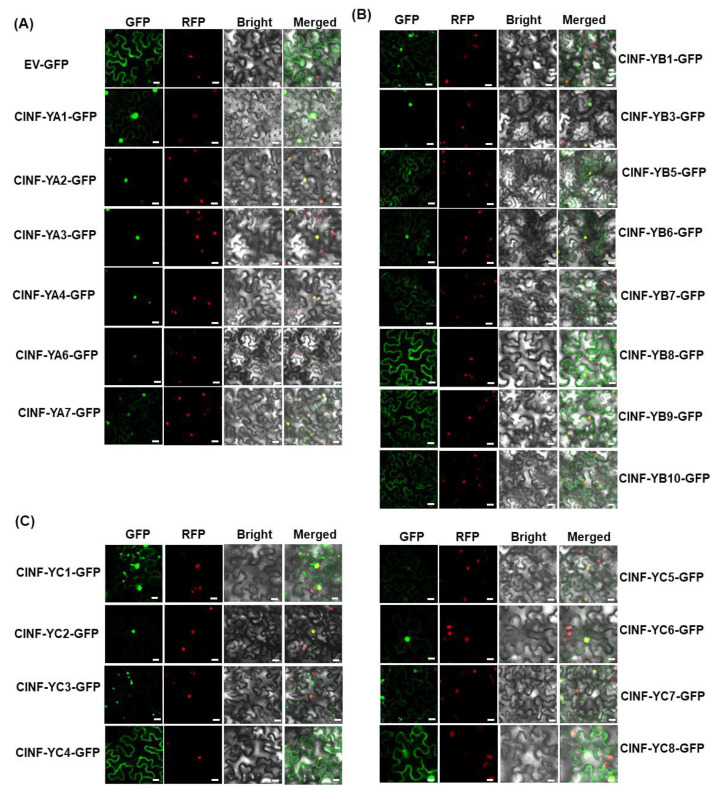
Subcellular localization of watermelon ClNF-Y proteins. (**A**) ClNF-YA subunits; (**B**) ClNF-YB subunits; (**C**) ClNF-YC subunits. Agrobacteria carrying pCAMBIA1300-ClNF-YAs-GFP, pCAMBIA1300-ClNF-YBs-GFP, pCAMBIA1300-ClNF-YCs-GFP, or pCAMBIA1300-GFP were infiltrated into leaves of *N. benthamiana* plants expressing a known nucleus-localized marker protein RFP-H2B. At 48 h after agroinfiltration, the GFP signal was visualized under a confocal laser scanning microscope in a dark field for green fluorescence (**left**) and red fluorescence (**middle left**), a white field for cell morphology (**middle right**), and in combination (**right**). Scale bars, 20 μm. Experiments were performed three times with similar results.

To explore the subcellular localization of the ClNF-Y proteins, agrobacteria carrying pCAMBIA1300-ClNF-YAs-GFP, pCAMBIA1300-ClNF-YBs-GFP, pCAMBIA1300ClNF-YCs-GFP, or pCAMBIA1300-GFP were infiltrated into the leaves of *Nicotiana benthamiana* plants expressing a red nuclear marker protein RFP-H2B [64]. The GFP signal from pCAMBIA1300-GFP-infiltrated leaves was distributed ubiquitously throughout the cells without specific compartmental localization (Figure 5A). The GFP signal from pCAMBIA1300-ClNF-YAs-GFP-infiltrated leaves was mainly observed in the nucleus, which was co-localized with the known nucleus marker RFP-H2B (Figure 5A). Similarly, a GFP signal from pCAMBIA1300-ClNF-YCs-GFP-infiltrated leaves was predominately seen in the nucleus, co-localized with RFP-H2B, except for pCAMBIA1300-ClNF-YC1-GFP, pCAMBIA1300-ClNF-YC4-GFP, pCAMBIA1300-ClNF-YC7-GFP, and pCAMBIA1300-ClNF-YC8-GFP, which were localized in the cells without specific compartmental localization (Figure 5C). By contrast, the GFP signal from pCAMBIA1300-ClNF-YBs-GFP-infiltrated leaves was detected throughout the cellular compartments including the nucleus, except for ClNF-YB3, which was mainly localized in the nucleus (Figure 5B). These data indicate that ClNF-YAs and ClNF-YCs are mainly targeted to the nucleus while ClNF-YBs are mostly localized in both nucleus and cytoplasm of the cells.

### 2.6. Interactions between the ClNF-Y Subunits and Assembly of the ClNF-Y Complexes

It has been reported that Arabidopsis AtNF-YBs and AtNF-YCs interact with each other to form heterodimers [23,25]. To explore the assembly of the ClNF-Y complexes, the interactions between ClNF-YBs and ClNF-YCs were first examined through a yeast two-hybrid (Y2H) system. However, some of the ClNF-YCs, such as ClNF-YC1, -YC2, and -YC4, showed autoactivation activity in Y2H assays even when a high concentration of AbA (500 ng/mL) was added to SD/-Trp plates (Figure S5). Therefore, bimolecular fluorescent complimentary (BiFC) assays were performed to examine the interactions between ClNF-YBs and ClNF-YCs. The results show that a YFP signal was observed in leaves co-expressing p2YN-ClNF-YB5/-YB6/-YB7/-YB8/-YB9/-YB10 and p2YC-ClNF-YC2/-YC3/-YC4/-YC5/-YC6/-YC7/-YC8, or co-expressing p2YN-ClNF-YB1/-YB3 and p2YC-ClNF-YC1, while no fluorescence was found in the negative controls (Figure S6). These data indicate that ClNF-YB5, -YB6, -YB7, -YB8, -YB9, and -YB10 interacted with ClNF-YC2, -YC3, -YC4, -YC5, -YC6, -YC7 and -YC8, and that ClNF-YB1 and -YB3 interacted with ClNF-YC1 (Table 2). Notably, ClNF-YC1 did not interact with ClNF-YB4, -YB5, -YB6, -YB7, -YB8, -YB9, and -YB10, while ClNF-YB1 and -YB3 did not interact with ClNF-YC2, -YC3, -YC4, -YC5, -YC6, -YC7, and -YC8 in BiFC assays (Table 2).

**Figure 6:**
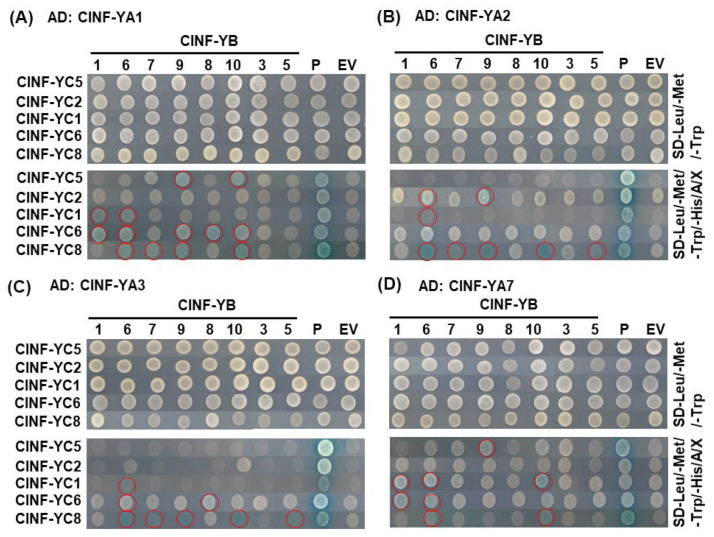
Assembly of the ClNF-Y complexes by the interactions of ClNF-YAs, ClNF-YBs, and ClNF-YCs in Y3H assays. ClNF-YAs were cloned into pGADT7 to fuse with the GAL4 activation domain, while paired combinations of ClNF-YBs and ClNF-YCs were cloned into pBridge to fuse with the GAL4 DNA-binding domain. Growth of yeasts co-transformed with indicated pGADT7 and pBridge vectors were grown on SD-Leu/-Met/-Trp noninteraction selective medium or on SD-Leu/-Met/-Trp/-His/AbA/X-α-gal interaction selective medium. Experiments were performed three times with similar results. Putative ClNF-Y complexes are indicated with open red circles.

To further explore the assembly of the ClNF-Y complexes, the interactions between ClNF-YAs and putative ClNF-YB/ClNF-YC heterodimers were investigated via yeast three-hybrid (Y3H) assays. For this purpose, ClNF-YBs and ClNF-YCs were inserted into pBridge vectors and ClNF-YAs were cloned into the pGADT7 vector. However, ClNF-YC1, -YC2, and -YC4 in combination with their interacting ClNF-YBs exhibited autoactivation activity in the pBridge vector when the yeast cells were grown on SD-Met/-Trp supplemented with 125 ng/mL AbA, and thus were not included further. In Y3H assays, the interaction of Arabidopsis AtNF-YA4 with the AtNF-YB3/-YC2 heterodimer, as previously reported [65], was included as a positive control. The results show that ClNF-YA1 interacted with the heterodimers of ClNF-YB9/-YC5, ClNF-YB10/-YC5, ClNF-YB1/-YC1, ClNF-YB6/-YC1, ClNF-YB1/-YC6, ClNF-YB6/-YC6, ClNF-YB9/-YC6, ClNF-YB8/-YC6, ClNF-YB10/-YC6, ClNF-YB6/-YC8, ClNF-YB7/-YC8, ClNF-YB9/-YC8, and ClNF-YB10/-YC8 (Figure 6A); ClNF-YA2 interacted with the heterodimers of ClNF-YB6/-YC2, ClNF-YB9/-YC2, ClNF-YB6/-YC1, ClNF-YB6/-YC8, ClNF-YB7/-YC8, ClNF-YB9/-YC8, ClNF-YB10/-YC8, and ClNF-YB5/-YC8 (Figure 6B); ClNF-YA3 interacted with the heterodimers of ClNF-YB6/-YC1, ClNF-YB6/-YC6, ClNF-YB8/-YC6, ClNF-YB6/-YC8, ClNF-YB7/-YC8, ClNF-YB9/-YC8, ClNF-YB10/-YC8, and ClNF-YB5/-YC8 (Figure 6C); and ClNF-YA7 interacted with the heterodimers of ClNF-YB9/-YC5, ClNF-YB1/-YC1, ClNF-YB6/-YC1, ClNF-YB10/-YC1, ClNF-YB1/-YC6, ClNF-YB6/-YC6, ClNF-YB6/-YC8, and ClNF-YB10/-YC8 (Figure 6D). However, ClNF-YA1, -YA2, -YA3, and -YA7 did not interact with any of the ClNF-YB3/-YC heterodimers (Figure 6). Similarly, ClNF-YA4 and ClNF-YA6 did not interact with any heterodimers of ClNF-YBs/-YCs (Figure S7). These data indicate that ClNF-YA1, ClNF-YA2, ClNF-YA3, and ClNF-YA7 can assemble into ClNF-Y complexes with different ClNF-YB/ClNF-YC heterodimers. Overall, a total of 37 putative ClNF-Y complexes (13 for ClNF-YA1/-YBs/-YCs, 8 for ClNF-YA2/-YBs/-YCs, 8 for ClNF-YA3/-YBs/-YCs, and 8 for ClNF-YA7/-YBs/-YCs) were identified. Notably, specificity in the assembly of ClNF-Y complexes was observed; for example, ClNF-YA2 assembled into the ClNF-Y complexes with the ClNF-YBs/-YC2 heterodimers while ClNF-YA1, -YA3, and -YA7 assembled into the ClNF-Y complexes with the ClNF-YBs/-YC8 heterodimers (Figure 6).

### 2.7. Expression Changes of ClNF-Y Genes in Response to Defense Hormones and a Fungal Pathogen

**Figure 7:**
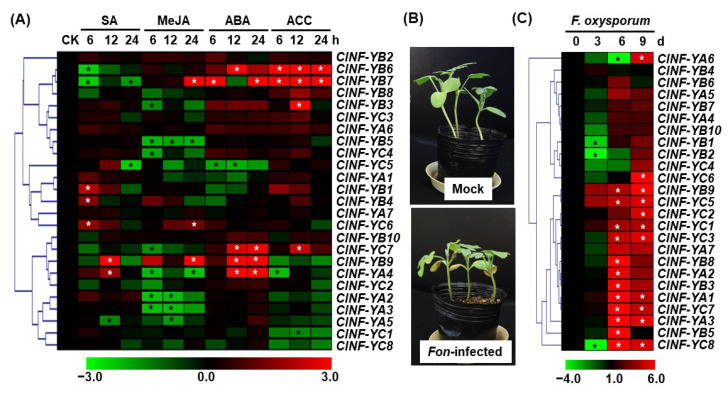
Expression changes of the watermelon *ClNF-Y* genes in response to hormones and *F. oxysporum* f. sp. *niveum*. (**A**) Expression changes of the *ClNF-Y* genes in response to SA, MeJA, ABA, and ACC. Four-week-old watermelon plants were treated by foliar spraying with 1 mM SA, 100 μM MeJA, 100 μM ABA, 100 μM ACC, or a similar volume of solution (as mock controls) and leaf samples were collected at indicated time points after treatment. (**B**) Disease phenotype of the Fon-inoculated plants at 11 d post-inoculation. (**C**) Expression changes in the *ClNF-Y* genes in the roots of watermelon plants after infection by *F. oxysporum* f. sp. *niveum*. Three-week-old plants were inoculated by dipping the roots in spore suspension (1 × 10^7^ spores/mL) of *F. oxysporum* f. sp. *niveum* or in mung bean liquid broth as mock-inoculated controls, and root samples were collected at indicated time points after inoculation. RT-qPCR was performed using the watermelon *ClGAPDH* gene as an internal control and relative expression levels are presented as log_2_ (FoldChange). Experiments were performed three times and data presented are the means from three independent experiments. * indicates the significant difference at the *p* < 0.01 level compared to the data in mock-treated (**A**) and mock-inoculated (**C**) plants at 0 h or 0 d, respectively.

To explore the involvement of *ClNF-Y* genes in disease resistance, expression patterns were analyzed via reverse transcription (RT)–quantitative polymerase chain reaction (qPCR) in watermelon plants after treatment with different stress hormones or infection by *Fon*, the causal agent of Fusarium wilt [59]. In plants treated with 1 mM SA, the expression of *ClNF-YA4*, *-YB1*, *-YB4*, *-YB9*, and *-YC6* was upregulated, while the expression of *ClNF-YA5, -YB6*, *-YB7*, and *-YC5* was downregulated compared to those in the untreated control plants (Figure 7A). The expression of *ClNF-YB7*, *-YB9*, and *-YC6* was upregulated, while the expression of *ClNF-YA2*, *-YA3*, *-YA4*, *-YA5*, *-YB3*, *-YB5*, *-YC4*, and *-YC7* was downregulated in plants after treatment with 100 μM MeJA, compared to control plants (Figure 7A). In plants treated with 100 μM ABA, the expression of *ClNF-YA4*, *-YB6*, *-YB7*, *-YB9*, and *-YC7* was upregulated, while the expression of *ClNF-YA1* and *ClNF-YC5* was downregulated, compared to control plants (Figure 7A). The expression of *ClNF-YB3*, *-YB6*, *-YB7*, and *-YC7* was upregulated, while the expression of *ClNF-YA4*, and *-YC1* was downregulated in plants after treatment with 100 μM ACC, compared to control plants (Figure 7A). Overall, most of the *ClNF-Y* genes were upregulated by SA, ABA, and ACC, and downregulated by MeJA (Figure 7A). Specifically, the expression of *ClNF-YB9* was upregulated by SA, MeJA and ABA, while the expression of *ClNF-YB7* was upregulated by MeJA, ABA, and ACC (Figure 7A). These data indicate that the *ClNF-Y* genes are responsive to different stress hormones and thus may be involved in distinct hormone-mediated signaling pathways in respect of stress response.

Because *Fon* is a vascular colonizing fungal pathogen that infects watermelon plants through the root system [59], the expression of *ClNF-Y* genes in root tissues was analyzed. Generally, typical leaf yellowing and wilting symptoms appear at ~7–10 d post-inoculation (dpi; Figure 7B), thus root samples were collected at 3, 6, and 9 dpi for analyzing the expression changes in the *ClNF-Y* genes after *Fon* infection. During the early stage of infection, the expression of most *ClNF-Y* genes was not affected, except that *ClNF-YB1*, *-YB2*, and *-YC8* were downregulated, at 3 dpi (Figure 7C). At 6 dpi during the colonization stage, the expression of *ClNF-YA1*, *-YA2*, *-YA3*, *-YB3*, *-YB5*, *-YB8*, *-YB9*, *-YC1*, *-YC3*, *-YC5*, *-YC7*, and *-YC8* was significantly upregulated, while the expression of *ClNF-YA6* was downregulated, in the roots of plants after *Fon* infection, compared to mock-inoculated plants (Figure 7C). At 9 dpi during the symptom appearance stage, the expression of *ClNF-YA1*, *-YA3*, *-YA6*, *-YB9*, *-YC1*, *-YC2*, *-YC3*, *-YC5*, *-YC6*, *-YC7*, and *-YC8* was remarkably upregulated by *Fon*, compared to mock-inoculated plants (Figure 7C). Overall, most of the *ClNF-Y* genes were upregulated in the roots of the watermelon plants after *Fon* infection (Figure 7C). These data indicate that most of the *ClNF-Y* genes respond to pathogen infection and thus may have functions in disease resistance against fusarium wilt.

### 2.8. Generation of ClNF-Y-Overexpressing Arabidopsis Lines and the Involvement of ClNF-Y in Growth and Development

**Figure 8:**
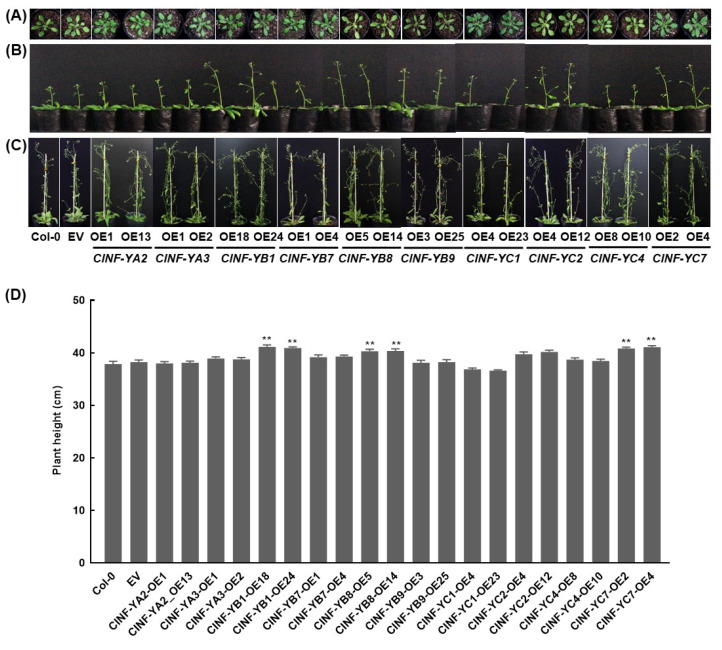
Growth and flowering phenotypes of the *ClNF-Y*-overexpressing plants. (**A–C**) Phenotypes of four-week-old plants before bolting (**A**), flowering (**B**), and six-week-old plants after bolting (**C**). (**D**) Plant heights of six-week-old plants. Experiments were performed three times with similar results. Data presented in (**D**) are the means ± SD and ** above the columns indicate significant differences at the *p* < 0.01 level.

To explore the biological functions of the *ClNF-Y* genes, 10 genes (*ClNF-YA2*, *-YA3*, *-YB1*, -*YB7, -YB8*, *-YB9*, *-YC1*, *-YC2*, *-YC4*, and *-YC7*), based on the assembly of ClNF-Y complexes (Figure 6) and the expression patterns (Figure 7), were selected to generate overexpression transgenic Arabidopsis lines through the floral dip method [66]. After hygromycin-resistance screening and genetic analyses, two homozygous transgenic lines with single-copy for each of the *ClNF-Y* genes (T3 generation) and similar expression levels of the transgenes were chosen for further studies. RT-qPCR analyses indicated that the *ClNF-Y* genes were transcribed normally in the transgenic Arabidopsis lines (Figure S8). Before bolting, the *ClNF-Y*-overexpressing Arabidopsis plants grew normally and were indistinguishable from the wild-type (WT) plants in terms of growth and morphological phenotype (Figure 8A). The *ClNF-YB1*-OE, *ClNF-YB8*-OE, *ClNF-YB9*-OE, *ClNF-YC2*-OE, and *ClNF-YC7*-OE plants flowered earlier by 2–4 d compared with the WT plants; the *ClNF-YA2*-OE, *ClNF-YA3*-OE, *ClNF-YB7*-OE, *ClNF-YC1*-OE, and *ClNF-YC4*-OE plants showed similar flowering to the WT plants (Figure 8B). After bolting, six-week-old *ClNF-YB1*-OE, *ClNF-YB8*-OE, and *ClNF-YC7*-OE plants were taller than the WT plants, while the plant heights of the other transgenic lines were comparable to the WT plants (Figure 8C,D). These data indicate that *ClNF-YB1*, *-YB8*, *-YB9*, *-YC2*, and *-YC7* play roles in flowering, and that *ClNF-YB1*, *-YB8*, and *-YC7* also function in vegetative growth.

### 2.9. Functions of the ClNF-Y Genes in Disease Resistance

**Figure 9:**
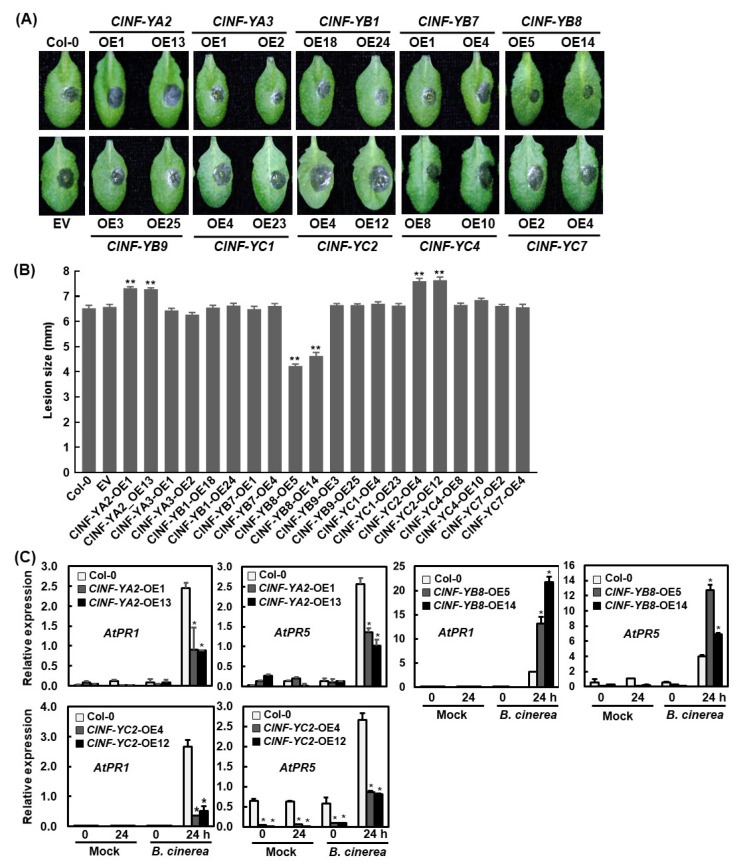
*Botrytis-cinerea*-caused disease phenotype on leaves from the *ClNF-Y*-overexpressing and WT Arabidopsis plants in detached leaf inoculation assays. (**A**) Disease symptoms and (**B**) lesion sizes. Fully expanded leaves detached from four-week-old plants were inoculated by dropping spore suspension (1 × 10^5^ spores/mL) of *B. cinerea* on leaf surface, and lesion sizes were measured at 3 dpi. Experiments were performed three times with similar results. (**C**) Expression changes of defense genes in WT and transgenic OE plants with or without infection of *B. cinerea*. Four-week-old plants were inoculated with *B. cinerea* and leaf samples were collected at 0 and 24 h post-inoculation. Data presented in (**B**,**C**) are the means ± SD and */** above the columns indicate significant differences at the *p* < 0.05 and *p* < 0.01 level, respectively.

To explore the possible functions of the *ClNF-Y* genes in disease resistance, the *ClNF-Y*-overexpressing Arabidopsis lines were assessed for their resistance phenotype against *Botrytis cinerea*, a necrotrophic fungus causing grey mold disease. When fully expanded leaves from four-week-old plants were inoculated with a drop of 3 μL spore suspension (2 × 10^5^ spores/mL), typical *B.-cinerea*-caused water-soaked necrotic lesions appeared at 2 dpi (Figure 9A). In repeated assays, the necrotic lesions on the detached leaves of the *ClNF-YA2*-OE and *ClNF-YC2*-OE plants were significantly larger, resulting in increases of approximately 12%, and 17%, respectively, while the necrotic lesions on the detached leaves of the *ClNF-YB8*-OE plants were remarkably smaller, leading to a reduction of ~32% in comparison to those on the WT leaves at 3 dpi (Figure 9A,B). Without infection of *B. cinerea*, the expression of *AtPR5* in *ClNF-YC2*-OE plants were markedly downregulated, while no significant change in the expression of *AtPR1* in *ClNF-YA2*-OE, *ClNF-YB8*-OE, and *ClNF-YC2*-OE plants and *AtPR5* in *ClNF-YA2*-OE and *ClNF-YB8*-OE plants was observed (Figure 9C). However, the expression levels of *AtPR1* and *AtPR5* were significantly downregulated in *ClNF-YA2*-OE and *ClNF-YC2*-OE plants but upregulated in *ClNF-YB8*-OE plants after infection of *B. cinerea* (Figure 9C). *B. cinerea*-caused necrotic lesions on the detached leaves of the *ClNF-YA3-OE*, *ClNF-YB1*-OE, *ClNF-YB7*-OE, *ClNF-YB9*-OE, *ClNF-YC1*-OE, *ClNF-YC4*-OE, and *ClNF-YC7*-OE plants were comparable to those on the WT leaves (Figure 9A,B). These data suggest that *ClNF-YB8* plays a positive role while *ClNF-YA2* and *-YC2* function negatively in terms of disease resistance against *B. cinerea* in transgenic Arabidopsis plants.

**Figure 10:**
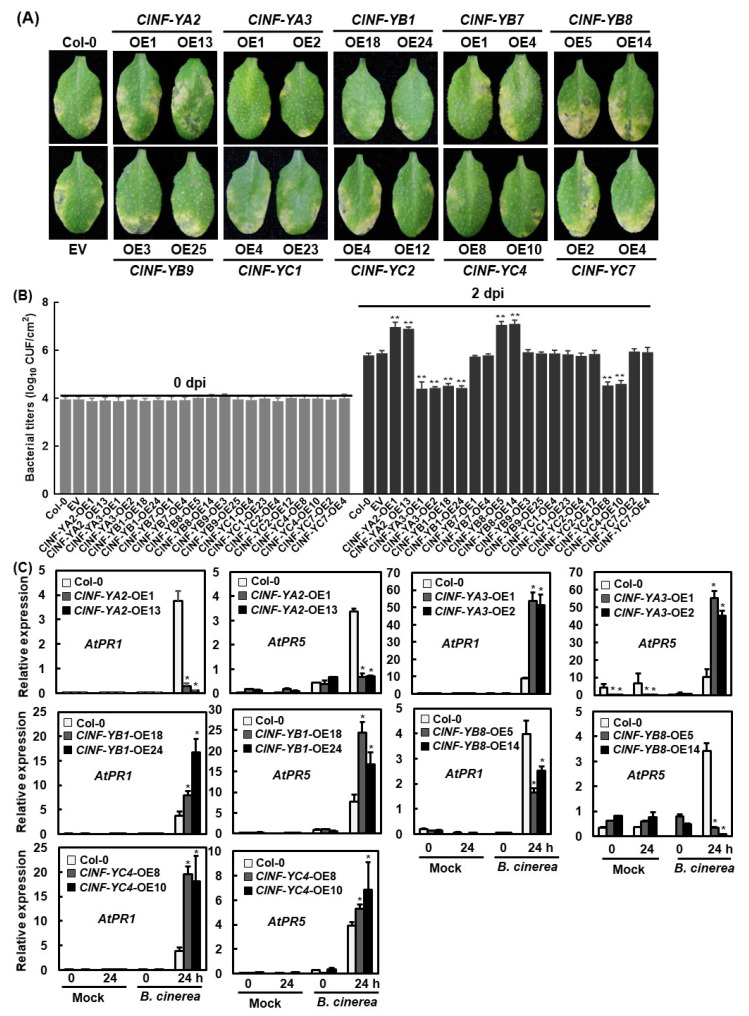
*Pseudomonas syringae* pv. *tomato* DC3000-caused disease phenotype on leaves of the *ClNF-Y*-overexpressing and WT Arabidopsis plants. (**A**) Disease symptoms and (**B**) bacterial growth titers in inoculated leaves. Fully expanded leaves of four-week-old plants were inoculated by injecting with *P. syringae* pv. *tomato* DC3000 suspension (OD_600_ = 0.0002) and photographed at 3 dpi. Leaf samples were collected at 0 and 2 dpi and bacterial growth in CFU/(cm^2^ leaf area) is shown. (**C**) Expression changes of defense genes in WT and transgenic OE plants with or without infection of *P. syringae* pv. *tomato* DC3000. Four-week-old plants were inoculated with *P. syringae* pv. *tomato* DC3000 and leaf samples were collected at 0 and 24 h post-inoculation. Experiments were performed three times with similar results. Data presented in (**B**,**C**) are the means ± SD and */** above the columns indicate significant differences at the *p* < 0.05 and *p* < 0.01 levels, respectively.

The function of *ClNF-Y* genes in disease resistance was further investigated through assessing the resistance phenotype of the *ClNF-Y*-overexpressing transgenic Arabidopsis plants against *Pseudomonas syringae* pv. *tomato* (*Pst*) DC3000, a hemibiotrophic bacterial pathogen causing leaf spot disease. When the Arabidopsis leaves were inoculated by injecting a bacterial inoculum of *Pst* DC3000, typical yellowing symptoms were observed on the inoculated leaves at 4 dpi (Figure 10A). Compared with those in the inoculated WT leaves, diseases on the inoculated leaves of the *ClNF-YA3*-OE, *ClNF-YB1*-OE, and *ClNF-YC4*-OE plants were reduced and these leaves supported less bacterial growth, resulting in decreases of 1.37, 1.26, and 1.22 orders of magnitude at 2 dpi (Figure 10A,B). By contrast, diseases on the inoculated leaves of *ClNF-YA2*-OE and *ClNF-YB8*-OE plants were much more severe, showing larger yellowing and necrotic areas, and these leaves supported more bacterial growth, leading to increases of 1.15 and 1.29 orders of magnitude at 2 dpi, as compared with those in the inoculated WT leaves (Figure 10A,B). Without infection of *Pst* DC3000, the expression of *AtPR1* and *AtPR5* in *ClNF-YA2*-OE, *ClNF-YA3*-OE, *ClNF-YB1*-OE, *ClNF-YB8*-OE, and *ClNF-YC4*-OE plants was not affected, except that the expression of *AtPR5* in *ClNF-YA3*-OE plants were markedly downregulated (Figure 10C). However, the expression levels of *AtPR1* and *AtPR5* were significantly upregulated in *ClNF-YA3*-OE, *ClNF-YB1*-OE, and *ClNF-YC4*-OE plants but downregulated in *ClNF-YA2*-OE and *ClNF-YB8*-OE plants after infection of *Pst* DC3000 (Figure 10C). In addition, disease symptoms on the inoculated leaves of *ClNF-YB7*-OE, *ClNF-YB9*-OE, *ClNF-YC1*-OE, *ClNF-YC2*-OE, and *ClNF-YC7*-OE plants were indistinguishable from those on the inoculated WT leaves (Figure 10A,B). These data indicate that *ClNF-YA3*, *-YB1*, and *-YC4* positively regulate while *ClNF-YA2* and *-YB8* negatively modulate the resistance of transgenic Arabidopsis plants against *Pst* DC3000.

## 3. Discussion

Unlike those in mammals and yeasts, the subunits of plant NF-Y complexes are encoded by relatively small gene families [20]. A Previous study has identified 19 *ClNF-Y* genes in the watermelon genome [60]. In the present study, further bioinformatics analyses identified a total 25 *ClNF-Y* genes in watermelon, among which 7 encode for *ClNF-YAs*, 10 for *ClNF-YBs*, and 8 for *ClNF-YCs* (Table 1), similar to those in cucumber [12], but fewer than those in Arabidopsis (36 *AtNF-Ys*), rice (34 *OsNF-Ys*), and tomato (59 *SlNF-Ys*) [10,11,16]. The *ClNF-YA* genes showed a highly complicated intron–exon organization with 4–7 introns, while the *ClNF-YB* and *ClNF-YC* genes exhibited variable intron–exon organizations (Figure 2B). Specifically, more than half of the *ClNF-YB* (5/10) and *ClNF-YC* (5/8) genes were intronless (Figure 2B), which is consistent with a universal feature of *NF-YB* and *NF-YC* genes in other plant species including cucumber [12]. Phylogenetic tree analysis revealed that the watermelon ClNF-Y proteins were closely related to those in Arabidopsis (Figure 1). These characteristics in phylogenetic relationships and gene structure imply the conserved feature of the evolution of the *NF-Y* families in plants. In the *ClNF-Y* family, only three segmentally duplicated genes were identified, and no tandemly duplicated gene was detected (Figure 3A), suggesting that segmental duplication is the major force driving the expansion of the *ClNF-Y* family in watermelon. This differs from that of the rice *OsNF-Y* and cucumber *CsNF-Y* families, whose expansions were driven by both segmental and tandem duplication events [11,12]. Furthermore, analyses of the interspecific syntenic relationship and the Ka/Ks ratios of the collinear *ClNF-Y* gene pairs with other plant species (Figure 3B; Tables S1 and S2) revealed that purifying selective pressure may have been a strong driving force in the evolution of the *ClNF-Y* family in watermelon.

The three subunits (NF-YA, NF-YB, and NF-YC) of the NF-Y complexes are generally recognized by the presence of conserved domains responsible for the interaction between the subunits and DNA binding to the CCAAT *cis*-element in the promoters of the target genes [6]. The ClNF-YAs contain NF-YB/YC interaction and DNA-binding domains, while the ClNF-YBs and ClNF-YCs harbor the HFM domains (Figure 2C, Figure S3). In addition, other conserved motifs were identified in ClNF-YAs and ClNF-YCs (Figure 2C). The structural features confer the basis for the subcellular localization, interaction, and biochemical activities of the ClNF-YAs, ClNF-YBs, and ClNF-YCs. For example, the ClNF-YAs and ClNF-YCs were predominately localized in nucleus (Figure 5A,C), which is consistent with the common knowledge that NF-YAs and NF-YCs contain NLSs and thus are generally localized to the nucleus [7,8,20,23]. Notably, ClNF-YC1, -YC4, -YC7, and -YC8 were found to localize in both the nucleus and cytoplasm of the cells, although they harbor NLSs similar to ClNF-YC2, -YC3, -YC5, and -YC6, which were predominately localized in nucleus, in the BiFC assays (Figure 5C). Because the NF-YBs and NF-YCs generally heterodimerize in the cytoplasm and then translocate into the nucleus [22,25,26], it is speculated that the difference in subcellular localization of ClNF-YCs may be due to their interactions with *N. benthamiana* NF-YBs. Unlike NF-YAs and NF-YCs, NF-YBs are not localized in the nucleus due to the lack of NLSs [19]. The majority of the ClNF-YBs were found to be localized in both the nucleus and cytoplasm of the cells without specific compartments (Figure 5B); however, ClNF-YB3 seemed to be localized in the nucleus (Figure 6B). Similar phenomena were also observed for cassava MeNF-YB11 and MeNF-YB16, and *Picea wilsonii* PwNF-YB3, which were mainly localized in the nucleus [58,67]. It is known that interactions and the formation of heterodimers with NF-YCs are essential for the translocation of NF-YBs from the cytoplasm into the nucleus [25,65]. In this regard, the nuclear localization of ClNF-YB3 (Figure 5B) may be due to the formation of heterodimers through interacting with unknown *N. benthamiana* NF-YCs in planta.

It is well known that NF-YBs and NF-YCs interact with each other in the cytoplasm to form heterodimers, which translocate into the nucleus and interact with NF-YAs to assemble heterotrimeric NF-Y complexes [5,22,25,26,28]. In the present study, a relationship between the subcellular localization and the interactions of ClNF-YAs, ClNF-YBs and ClNF-YCs was noted. For example, ClNF-YC2 and -YC6 were localized in the nucleus and interacted with ClNF-YB6, -YB8, -YB9, and -YB10, and the heterodimers formed by these ClNF-YBs and ClNF-YCs assembled into ClNF-Y complexes with ClNF-YA1, -YA2, and -YA7 (Figure 5 and Figure 6, Table 2). How many heterotrimeric complexes could be formed by the combined subunits in the NF-Y family is a critical issue in terms of understanding the molecular mechanism of NF-Ys in plants. Protein–protein interaction analysis in BiFC assays indicated specific interactions between ClNF-YBs and ClNF-YCs; for example, ClNF-YB5, -YB6, -YB7, -YB8, -YB9, and -YB10 interacted with most of the ClNF-YCs except ClNF-YC1, while ClNF-YB1 and -YB3 interacted with ClNF-YC1 (Table 2; Figure S6). This is consistent with the observation that, in the Arabidopsis AtNF-Y family, AtNF-YBs and AtNF-YCs could interact in many combinations and form different heterodimers [23,25]. However, no interactions of ClNF-YC1 with ClNF-YB4, -YB5, -YB6, -YB7, -YB8, -YB9, and -YB10, and of ClNF-YB1 and -YB3 with ClNF-YC2, -YC3, -YC4, -YC5, -YC6, -YC7, and -YC8 were detected (Table 2). This is unlikely due to the failure in the expression of the fusion proteins in *N. benthamiana* leaves because all these ClNF-YBs and ClNF-YCs were found to interact with other ClNF-YCs and ClNF-YBs, respectively, in the same BiFC assays (Table 2; Figure S6). Given that the ClNF-Y family contains 7 *ClNF-YAs*, 10 *CLNF-YBs*, and 8 *ClNF-YCs* (Table 1), theoretically, there should be more than 500 heterotrimeric combinations. Approximately 1000 heterotrimeric combinations were estimated for the Arabidopsis *AtNF-Y* family with 36 members [20]. A total of 37 putative heterotrimeric ClNF-Y complexes were identified in Y3H assays (Figure 6). This number was far below the theoretically estimated number (240 complexes) when 4 ClNF-YAs, 8 ClNF-Ybs, and 5 ClNF-YCs were included (Figure 6). Although the interactions between ClNF-YB6 and ClNF-YC1, ClNF-YB1 and ClNF-YC6, and ClNF-YB10 and ClNF-YC1 were not detected in BiFC assays (Table 2; Figure S6), they did assemble into ClNF-Y complexes with ClNF-YA1, ClNF-YA2, ClNF-YA3, or ClNF-YA7 in Y3H assays (Figure 6), probably due to different techniques used in the experiments. Furthermore, ClNF-YA4 and -YA6 did not interact with any of the ClNF-YBs/ClNF-YCs heterodimers (Figure S7). The lower number of ClNF-Y complexes identified may be due to (1) specific interactions between ClNF-YBs and ClNF-YCs, as described above; (2) growth, developmental, and environmental cue-mediated interactions between the subunits and/or assembly of the complexes; and (3) transient and highly dynamic assembly of many complexes in vivo [28]. Therefore, further in-depth characterization of the heterotrimeric ClNF-Y complexes will provide insights into the biochemical and molecular mechanisms for the functions of the *ClNF-Y* family.

The functions of NF-Ys in plant growth and development have been well documented [6,28]. Bioinformatics analysis identified plenty light-responsive, growth, and development-associated *cis*-elements in promoters of the *ClNF-Y* genes (Figure 4; Figure S4), implying their involvement in the regulation of growth and development. This is directly supported by the observations that the *ClNF-YB1*-OE, *ClNF-YB8*-OE, *ClNF-YB9*-OE, *ClNF-YC2*-OE, and *ClNF-YC7*-OE plants showed an earlier flowering phenotype and that the *ClNF-YB1*-OE, *ClNF-YB8*-OE, and *ClNF-YC7*-OE plants grew taller after bolting (Figure 8). ClNF-YB1, -YB8, and -YC2 are phylogenetically related to AtNF-YB3, -YB9, and -YC2, respectively (Figure 1), which play positive roles in the promotion of flowering in Arabidopsis [68,69]. Notably, ClNF-YB1, -YB8, and -YC7 exhibited a pleiotropic effect on flowering and growth in transgenic Arabidopsis. Moreover, 5 of the 10 *ClNF-Y* genes selected for functional studies in transgenic Arabidopsis conferred an earlier flowering phenotype, indicating the wide involvement of the *ClNF-Y* family in the regulation of flowering time. Detailed examination of the phenotypes (e.g., seed setting, size, and weight) of the *ClNF-Y*-overexpressing Arabidopsis lines, especially *ClNF-YB1-OE*, *ClNF-YB8*-OE, and *ClNF-YC7*-OE lines, will provide further understanding on the functions and mechanism of the *ClNF-Y* genes in growth and development.

The plant NF-Y family has also been implicated in abiotic and biotic stress responses [6,28]. The fact that a large number of hormone- and stress-responsive *cis*-elements are present in the promoter regions (Figure 4) suggests the involvement of the *ClNF-Y* family in stress response. In particular, the *ClNF-YB7* and *-YC7* promoters harbor ABA-responsive element ABRE (Figure 4), and accordingly, the expression of *ClNF-YB7* and *-YC7* was upregulated by ABA (Figure 7A), implying their involvement in abiotic stress response. This is partially supported by the observation that Arabidopsis AtNF-YC2, closely related to ClNF-YC7 (Figure 1), has been implicated in the regulation of stress genes in Arabidopsis [65]. However, the presence of the SA-responsive TCA element in 13 promoters of the *ClNF-Y* genes, the MeJA-responsive element CGTCA-motif in 12 promoters, and the ethylene-responsive element ERE in 14 promoters (Figure 4), implies that these *ClNF-Y* genes may participate in the SA-, JA-, and ethylene-mediated signaling pathways and thus play roles in disease resistance in watermelon. The presence of defense-hormone-responsive *cis*-elements in the promoters seems to be consistent with the expression changes in the *ClNF-Y* genes in watermelon plants after treatment with SA, MeJA, or ACC. For instance, the *ClNF-YB9* promoter contains three MeJA-responsive CGTCA-motifs (Figure 4); accordingly, the expression of *ClNF-YB9* was induced by MeJA (Figure 7A). Similarly, the *ClNF-YB6* and *-YB7* promoters harbored three and two ethylene-responsive ERE elements, respectively (Figure 4), and their expression was strongly upregulated by ACC (Figure 7A). Furthermore, the majority of the *ClNF-Y* genes were induced by *Fon*, and the pathogen-induced expression was highly evident in roots (Figure 7C). Among these pathogen-inducible *ClNF-Y* genes, the *ClNF-YA1*, *-YB8*, and *-YB9* promoters harbor a pathogen-inducible Box S element (Figure 4), which is known to confer a high level of expression of the target genes in response to elicitors, oomycetes, and bacterial pathogens [70,71,72]. Functional studies in transgenic Arabidopsis revealed that 6 *ClNF-Y* genes play a role in disease resistance (Figure 9 and Figure 10). Specifically, *ClNF-YA2* and *-YC2* negatively regulated while *ClNF-YB8* positively regulated resistance against *B. cinerea* in transgenic Arabidopsis plants (Figure 9). *ClNF-YA2* and *-YB8* negatively regulated while *ClNF-YA3*, *-YB1*, and *-YC4* positively modulated resistance against *Pst* DC3000 in transgenic Arabidopsis plants (Figure 10). The alterations in resistance against *B. cinerea* and *Pst* DC3000 were accompanied with the changes in the pathogen-induced expression of defense genes in *ClNF-YA2*-OE, *ClNF-YA3*-OE, *ClNF-YB1*-OE, *ClNF-YB8*-OE, *ClNF-YC2*-OE, and *ClNF-YC4*-OE plants (Figure 9 and Figure 10). Notably, overexpression of *ClNF-YA2* in transgenic Arabidopsis resulted in attenuated resistance against both *B. cinerea* and *Pst* DC3000; however, overexpression of *ClNF-YB8* led to opposite functions in resistance against these two pathogens, e.g., enhanced resistance against *B. cinerea* but attenuated resistance against *Pst* DC3000 (Figure 9 and Figure 10). Generally, the defense response against (hemi)biotrophic pathogens such as *Pst* DC3000 is modulated through SA signaling, while resistance against necrotrophic pathogens like *B. cinerea* is regulated by JA/ET signaling [73,74]. There are both antagonistic and synergistic interactions between the SA and JA/ET signaling pathways to enable plants to activate appropriate defense responses against different invading pathogens [73,75,76]. In this regard, it is therefore likely that *ClNF-YA2* and *-YB8* function in disease resistance through different mechanisms. Further characterization of the target genes will provide insights into the molecular mechanisms by which key subunits or the ClNF-Y complexes regulate disease resistance in plants. Furthermore, the fact that 6 of the 10 functionally studied *ClNF-Y* genes played roles in disease resistance (Figure 9 and Figure 10) indicates the importance of the *ClNF-Y* family in plant disease resistance. This is similar to the recent observation that overexpression of 3 out of 11 rice *OsNF-YA* genes significantly affected susceptibility to two viral pathogens [55]. Therefore, further functional studies on the remaining *ClNF-Y* genes will be helpful to provide comprehensive understanding of the involvement of the *ClNF-Y* family in disease resistance.

## 4. Materials and Methods

### 4.1. Plant Materials, Growth Conditions, and Treatments

Watermelon (*Citrullus lanatus* L., cv. Zaojia), Arabidopsis WT and transgenic lines, and *N. benthamiana* plants were grown in a soil mix (clay: soil = 3:1) in a growth room with a 14 h light/10 h dark cycle under fluorescent light (200 μE m^2^ s^−1^) at 22–24 °C and 70% relative humidity (RH). Arabidopsis seedlings were grown on 1/2 MS plates at 22 °C with 75% RH under a 16 h light/8 h dark cycle for 7 d and then transplanted to a soil mix (clay:soil = 1:1) in a growth room under a 16 h light/8 h dark cycle at 22 °C and 75% humidity for normal growth or under an 8 h light/16 h dark cycle for disease assays. Hormone treatment was applied on four-week-old watermelon plants by foliar spraying with 1 mM SA (Sigma-Aldrich, St. Louis, MO, USA), 100 μM MeJA (Sigma-Aldrich, St. Louis, MO, USA), 100 μM ABA (Sigma-Aldrich, St. Louis, MO, USA), 100 μM ACC (Sigma-Aldrich, St. Louis, MO, USA), or an equal volume of solution containing only 0.1% ethanol and 0.02% Tween20 as controls. Pathogen inoculation was performed on three-week-old watermelon plants by the root-dipping inoculation method as previously described [77]. Spore inoculum of *Fon* race 1 strain ZJ1 (1 × 10^7^ spores/mL) was prepared as previously described [78]. The main roots of the watermelon plants were cut up to one-third, and then dipped for 15 min in spore inoculum of *Fon* or in medium broth as mock-inoculated controls. The inoculated plants were replanted in soil and allowed to grow in the same growth room as described above. Root and leaf samples were collected at indicated time points after treatment/inoculation, frozen in liquid nitrogen, and stored at −80 °C until use.

### 4.2. Identification and Bioinformatics Analysis of the Watermelon ClNF-Y Family

Arabidopsis AtNF-Ys protein sequences were downloaded from TAIR (https://www.arabidopsis.org, accessed on 11 May 2022) based on a previous report [10] and were used as queries to search via the BLASTp program for putative *ClNF-Y* genes and proteins in the watermelon genome in the Cucurbit Genomics Databases (http://cucurbitgenomics.org/organism/21 for 97,103 v2 and http://cucurbitgenomics.org/organism/4 for cv. Charleston Gray, accessed on 11 May 2022) [61,62]. The putative ClNF-Ys protein sequences were examined by domain analysis programs PFAM (http://pfam.sanger.ac.uk/, accessed on 13 May 2022) (PF02045 and PF00808) and SMART (http://smart.emblheidelberg.de/, accessed on 13 May 2022) with default cutoff parameters. The protein properties of ClNF-Ys, such as the number of amino acids, molecular weights, and isoelectric points (*p*I) were predicted on the ExPASy Proteomics Server (http://expasy.org/, accessed on 15 May 2022). Sequence alignment was carried out using the Clustal X1.8 program, and a phylogenetic tree was constructed using the pairwise gap deletion option, Poisson model, and 1000 bootstrap replicates in MEGA version 7.0 (https://www.megasoftware.net, accessed on 20 May 2022). Putative conserved motifs in the ClNF-Y proteins were characterized using the Multiple Em for Motif Elicitation program (MEME, http://www.meme.sdsc.edu/meme/meme.html, accessed on 20 May 2022) with optimized parameter settings: repetition number, any; minimum motif width, 6; maximum motif width, 50; maximum number of motifs, 20 [79].

A gene structure featuring introns and exons in the predicted *ClNF-Y* genes was constructed using Gene Structure Display Server 2.0 (GSDS) (http://gsds.cbi.pku.edu.cn/, accessed on 21 May 2022) [80]. The MCScanX algorithm with default parameters [81] was used to scan orthologous regions containing the watermelon *ClNF-Y* genes, and the corresponding plot was created using Dual Synteny Plot for MCscanX in TBtools software version 1.1044 [82]. The chromosomal localization of the *ClNF-Y* genes was obtained in the watermelon genome database (http://cucurbitgenomics.org/organism/21, accessed on 23 May 2022) and visualized using MapChart software (https://www.wur.nl/en/show/Mapchart.htm, accessed on 23 May 2022) [83]. The synteny relationships of the orthologous *NF-Y* genes between watermelon and other selected species (Arabidopsis, rice, and cucumber) were visualized using the Advance Circos package of TBtools [83]. DnaSP software version 6 was used to calculate the nonsynonymous (Ka)/synonymous (Ks) values of the duplicated *ClNF-Y* gene pairs [84]. The Plant CARE database (http://bioinformatics.psb.ugent.be/webtools/plantcare/html/, accessed on 25 May 2022) was employed to predict the putative *cis*-elements in the 1500 bp promoter regions of the *ClNF-Y* genes [63].

### 4.3. Cloning of ClNF-Y Genes

Total RNA was extracted using Trizol reagent and treated with RNase-free DNase (Takara, Tokyo, Japan) according to the manufacturer’s instructions. First-strand cDNA was synthesized using AMV reverse transcriptase (Takara, Tokyo, Japan) with oligo d(T) primer according to the manufacturer’s instructions. The coding sequences for *ClNF-Y* genes were amplified using gene-specific primers (Table S4) and cloned into pMD19-T vector (Takara, Tokyo, Japan) via T/A cloning, yielding pMD19-ClNF-YAs, pMD19-ClNF-YBs, and pMD19-ClNF-YCs, which were confirmed by sequencing.

### 4.4. Subcellular Localization Assays

The coding sequences of the *ClNF-Y* genes were amplified using gene-specific primers (Table S4) and inserted into pCAMBIA1300s, generating pCAMBIA1300s-ClNF-Ys-GFPs, which were then transformed into *Agrobacterium tumefaciens* strain GV3101. Agrobacteria carrying pCAMBIA1300s-ClNF-Ys-GFP or pCAMBIA1300s-GFP were infiltrated into leaves of *N. benthamiana* plants expressing the RFP-H2B marker [64]. The GFP signal was excited at 488 nm and detected under a Zeiss LSM780 confocal laser scanning microscope (Zeiss, Oberkochen, Germany) using a 500–530 nm emission filter, at 48 h after agroinfiltration.

### 4.5. BiFC Assays

The coding sequences of the *ClNF-YBs* or *ClNF-YCs* were amplified using gene-specific primers (Table S4) and inserted into p2YN and p2YC vectors, respectively, yielding p2YN-ClNF-YBs and p2YC-ClNF-YCs, which were then transformed into *Agrobacterium tumefaciens* strain GV3101. Agrobacteria harboring different pairs of p2YN-ClNF-YB and p2YC-ClNF-YC plasmids were infiltrated into leaves of *N. benthamiana* plants expressing the RFP-H2B marker [64]. YFP and RFP signals were detected under a Zeiss LSM780 confocal laser scanning microscope (Zeiss, Oberkochen, Germany), at 48 h after agroinfiltration.

### 4.6. Y3H Assays

The Y3H assay was performed using the Matchmaker GAL4 Two-Hybrid System according to the manufacturer’s recommendations (Clontech, Mountain View, CA, USA). The coding sequences of *ClNF-YBs* and *ClNF-YCs* were inserted into pBridge, forming pBridge-ClNF-YB/ClNF-YC constructs, and the coding sequences of *ClNF-YAs* were cloned into pGADT7, yielding pGADT7-ClNF-YAs. Plasmid pBridge-ClNF-YBs/ClNF-YCs and pGADT7-ClNF-YAs were co-transferred into yeast strain Y2HGold and plated on SD-Leu/-Met/-Trp medium. Colonies were transferred to the appropriate SD-Leu/-Met/-Trp/-His/AbA/X-α-gal selective medium. Specific activities of β-galactosidase were detected according to the manufacturer’s instructions. Arabidopsis AtNF-YA4, AtNF-YB3, and AtNF-YC2 were used as positive controls [65].

### 4.7. RT-qPCR Assays

Total RNA was extracted using Trizol reagent (Invitrogen, Carlsbad, CA, USA) according to the manufacturer’s instructions, and then reverse-transcribed into cDNA using the HiScript QRT SuperMix kit (Vazyme, Nanjing, China). Each qPCR contained 12.5 μL of AceQ qPCR SYBR Green Master Mix (Vazyme, Nanjing, China), 0.1 μg of cDNA, and 7.5 pmol of each gene-specific primer (Table S4) in a final volume of 25 μL. The qPCR was performed in a CFX96 real-time PCR detection system (Bio-Rad, Hercules, CA, USA). Watermelon *ClGAPDH* was used as an internal control and relative gene expression levels were calculated using the 2^−△△CT^ method. Data were normalized with those in mock-treated or mock-inoculated plants at each time point. Primers used are listed in Table S4.

### 4.8. Generation and Characterization of ClNF-Y-Overexpressing Transgenic Lines

Arabidopsis transformation with agrobacteria carrying pCAMBIA1300s-ClNF-Ys-GFP was performed using the floral dip method [66]. Putative positive transgenic plants from T1 seeds were selected on 1/2 MS medium containing 50 μg/mL hygromycin. Single-copy transgenic lines and homozygous lines were obtained by screening for a 3:1 segregation ratio of hygromycin-resistant character and 100% hygromycin-resistant phenotype in T2 and T3 generations on 1/2 MS medium supplemented with 50 μg/mL hygromycin, respectively.

### 4.9. Disease Assays

Disease assays with *B. cinerea* were performed as previously described [85]. *B. cinerea* was grown on 2 × V_8_ (36% V_8_ juice, 0.2% CaCO_3_, 2% agar) medium at 25 °C for 8~10 d, and spores were collected, and then resuspended in a 4% maltose and 1% peptone buffer to a final concentration of 2 × 10^5^ spores/mL. Fully expanded leaves were detached from four-week-old Arabidopsis plants and inoculated by dropping 3 μL spore suspension. Disease was estimated by measuring the lesion sizes.

*P. syringae* pv. *tomato* DC3000 was grown in King’s B broth and collected by centrifugation, followed by re-suspending in 10 mM MgCl_2_ solution to OD_600_ = 0.002. Inoculation was performed by hand infiltration using 1 mL syringes without needles into rosette leaves of four-week-old Arabidopsis plants, as described previously [86]. Leaf discs from inoculated leaves were collected and homogenized in 10 mM MgCl_2_ to quantify in planta bacterial growth.

### 4.10. Statistical Analysis

All the experiments were performed independently at least three times. The data obtained were subjected to statistical analysis according to Student’s *t*-test, and the probability values of *p* < 0.05 or *p* < 0.01 were considered as significant difference between different treatments.

## 5. Conclusions

In the present study, the watermelon *ClNF-Y* family was re-characterized and a total of 25 family members (7 *ClNF-YAs*, 10 *CLNF-YBs*, and 8 *ClNF-YCs*) were identified, further enlarging the family by adding 6 new members [60]. Structural features of genes and proteins, phylogenetic and syntenic relationships, *cis*-elements in promoters, subcellular localization, assembly of the ClNF-Y complexes, expression changes in response to defense hormones and pathogen infection, and putative functions in disease resistance were comprehensively investigated. A total of 37 putative ClNF-Y complexes that were assembled by ClNF-YA1, -YA2, -YA3, and -YA7 with diverse ClNF-YB/-YC heterodimers were identified. Expression analysis revealed that most of the *ClNF-Y* genes responded with distinct patterns to defense hormones and infection of a vascular-infecting pathogen, *F. oxysporum* f. sp. *niveum*. Functional studies in transgenic Arabidopsis revealed that 6 *ClNF-Y* genes (*ClNF-YA2*, *-YA3*, *-YB1*, *-YB8*, *-YC2*, and *-YC4*) played roles in disease resistance. It should be noted, however, that the functional analysis in the present study was performed via ectopic overexpression of the *ClNF-Y* genes in Arabidopsis, and the intrinsic functions of the *ClNF-Y* genes, especially those having a disease resistance function in transgenic Arabidopsis, need to be further investigated in watermelon disease resistance through overexpression and CRISPR/Cas9-based knockout approaches. The re-characterization of the *ClNF-Y* family provides a foundation from which to investigate the biological function of *ClNF-Y* genes in terms of growth, development, and stress response in watermelon, and the identification of the functions of some *ClNF-Y* genes in disease resistance enables further exploration of the molecular mechanism of *ClNF-Ys* in regulating watermelon disease resistance.

## Figures and Tables

**Table 1:** Information on the watermelon *ClNF-Y* genes.

Subunits	Genes	Locus ID (97103 v2)	Chromosomes	ORF (bp)	Protein (aa)	MW (Da)	*p*I
*ClNF-YA*	*ClNF-YA1*	Cla97C01G025170	1	666	221	24,444.14	8.27
	*ClNF-YA2*	Cla97C02G049270	2	1230	409	44,283.62	6.49
	*ClNF-YA3*	Cla97C03G063440	3	810	269	29,487.43	6.97
	*ClNF-YA4*	Cla97C04G068610	4	816	271	30,768.39	9.08
	*ClNF-YA5*	Cla97C09G166390	9	954	317	35,487.74	8.47
	*ClNF-YA6*	Cla97C10G201180	10	972	323	35,228.14	9.14
	*ClNF-YA7*	Cla97C11G207940	11	1044	347	38,318.83	7.73
*ClNF-YB*	*ClNF-YB1*	Cla97C02G035590	2	618	205	20,920.07	6.61
	*ClNF-YB2*	Cla97C02G039490	2	525	174	18,999.25	5.23
	*ClNF-YB3*	Cla97C02G048360	2	486	161	17,972.03	5.0
	*ClNF-YB4*	Cla97C03G067370	3	405	134	15,544.21	4.67
	*ClNF-YB5*	Cla97C06G124610	6	576	191	21,345.08	4.57
	*ClNF-YB6*	Cla97C07G136140	7	678	225	24,739.47	7.98
	*ClNF-YB7*	Cla97C09G168740	9	480	159	17,290.24	5.08
	*ClNF-YB8*	Cla97C10G188720	10	528	175	18,844.55	5.73
	*ClNF-YB9*	Cla97C10G188900	10	672	223	24,150.34	6.01
	*ClNF-YB10*	Cla97C10G203800	10	522	173	18,705.82	6.35
*ClNF-YC*	*ClNF-YC1*	Cla97C02G047110	2	603	280	22,498.46	9.17
	*ClNF-YC2*	Cla97C02G048620	2	843	260	31,251.64	4.77
	*ClNF-YC3*	Cla97C03G067230	3	783	220	28,842.77	6.16
	*ClNF-YC4*	Cla97C06G127670	6	663	117	24,223.41	4.97
	*ClNF-YC5*	Cla97C07G135150	7	354	140	12,986.36	7.96
	*ClNF-YC6*	Cla97C08G152900	8	423	266	15,794.05	8.99
	*ClNF-YC7*	Cla97C09G176240	9	801	283	29,969.79	6.33
	*ClNF-YC8*	Cla97C11G218950	11	852	280	31,695.19	5.09

**Table 2:** Summary of the interactions between ClNF-YBs and ClNF-YCs in BiFC assays.

				ClNF-YBs					
		1	3	5	6	7	8	9	10
**ClNF-YCs**	1	+	+	−	−	−	−	−	−
	2	−	−	+	+	+	+	+	+
	3	−	−	+	+	+	+	+	+
	4	−	−	+	+	+	+	+	+
	5	−	−	+	+	+	+	+	+
	6	−	−	+	+	+	+	+	+
	7	−	−	+	+	+	+	+	+
	8	−	−	+	+	+	+	+	+

+, positive; −, negative.

## Data Availability

Not applicable.

## Supplementary Materials

The supporting information can be downloaded at https://www.mdpi.com/article/10.3390/ijms232415778/s1.
