# Supplementary material for: The NF-Y Transcription Factor Family in Watermelon: Re-Characterization, Assembly of ClNF-Y Complexes, Hormone- and Pathogen-Inducible Expression and Putative Functions in Disease Resistance
Source: Int J Mol Sci. 2022 Dec 12;23(24):15778. doi: 10.3390/ijms232415778 (PMC9778975; doi:10.3390/ijms232415778)
Supplement: Supplementary file 1 [file ijms-23-15778-s001.zip › ijms-2030950-supplementary.pdf]

Supplementary Materials

The NF-Y Transcription Factor Family in Watermelon: Re-Characterization, Assembly of ClNF-Y Complexes, Hormone- and Pathogen-Inducible Expression and Putative Functions in Disease Resistance

Siyu Jiang, Hui Wang, Ya Wen, Jiayu Liang, Dayong Li * and Fengming Song *

Key Laboratory of Crop Diseases and Insect Pests of Ministry of Agriculture, Institute of Biotechnology, Zhejiang University, Hangzhou 310058, China
* Correspondence: dyli@zju.edu.cn (D.L.); fmsong@zju.edu.cn (F.S.)

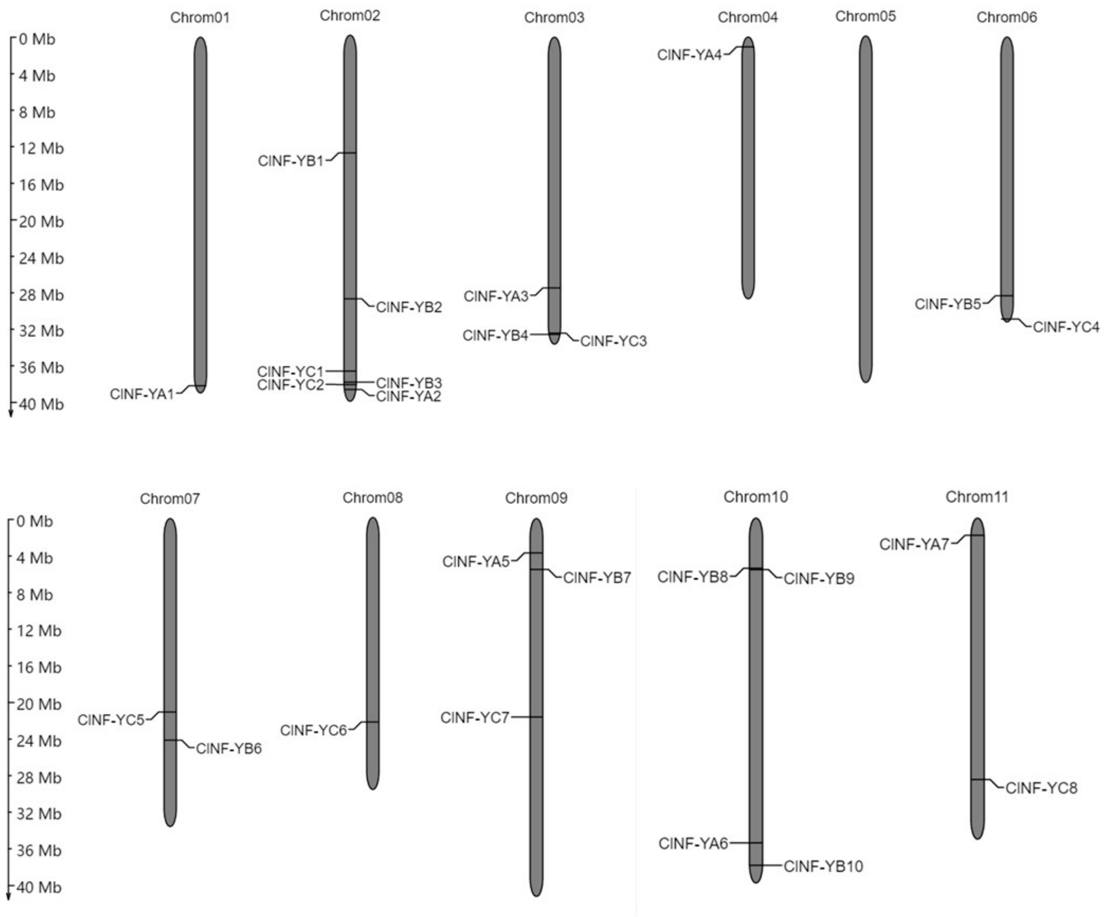

Figure S1. Distribution of *ClNF-Y* genes on watermelon chromosomes.

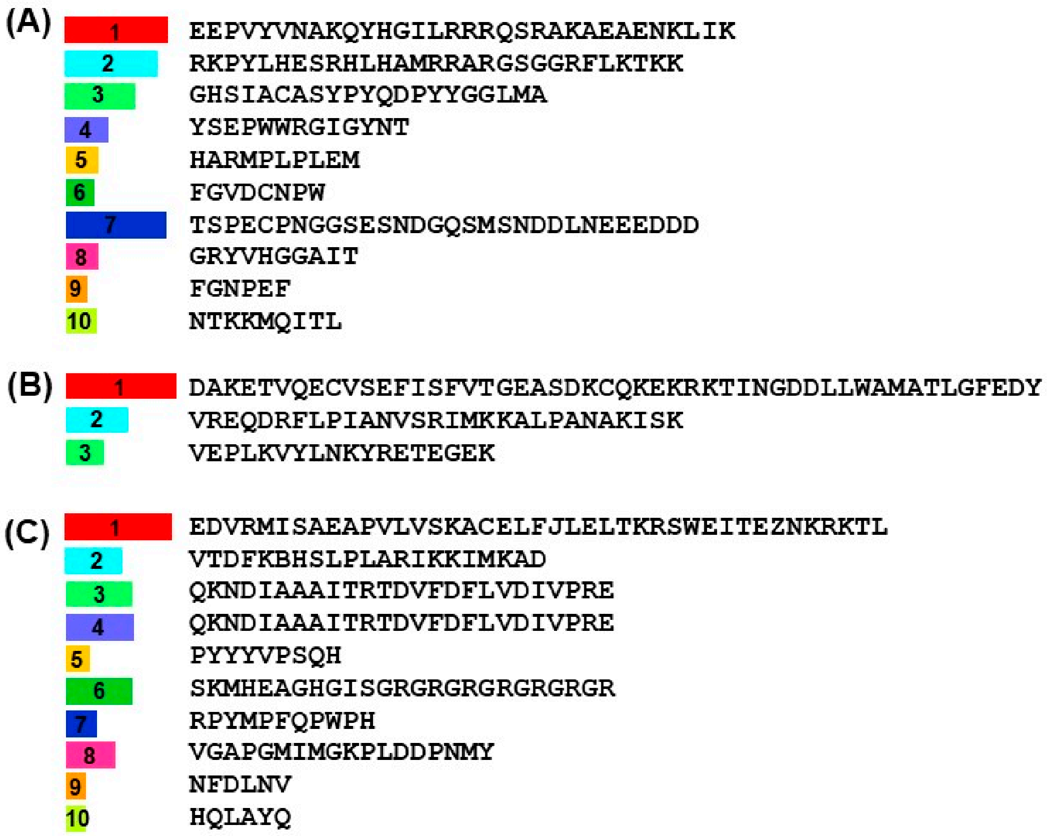

Figure S2. Conserved motifs in ClNF-YAs (A), ClNF-YBs (B), and ClNF-YCs (C) identified by MEME.

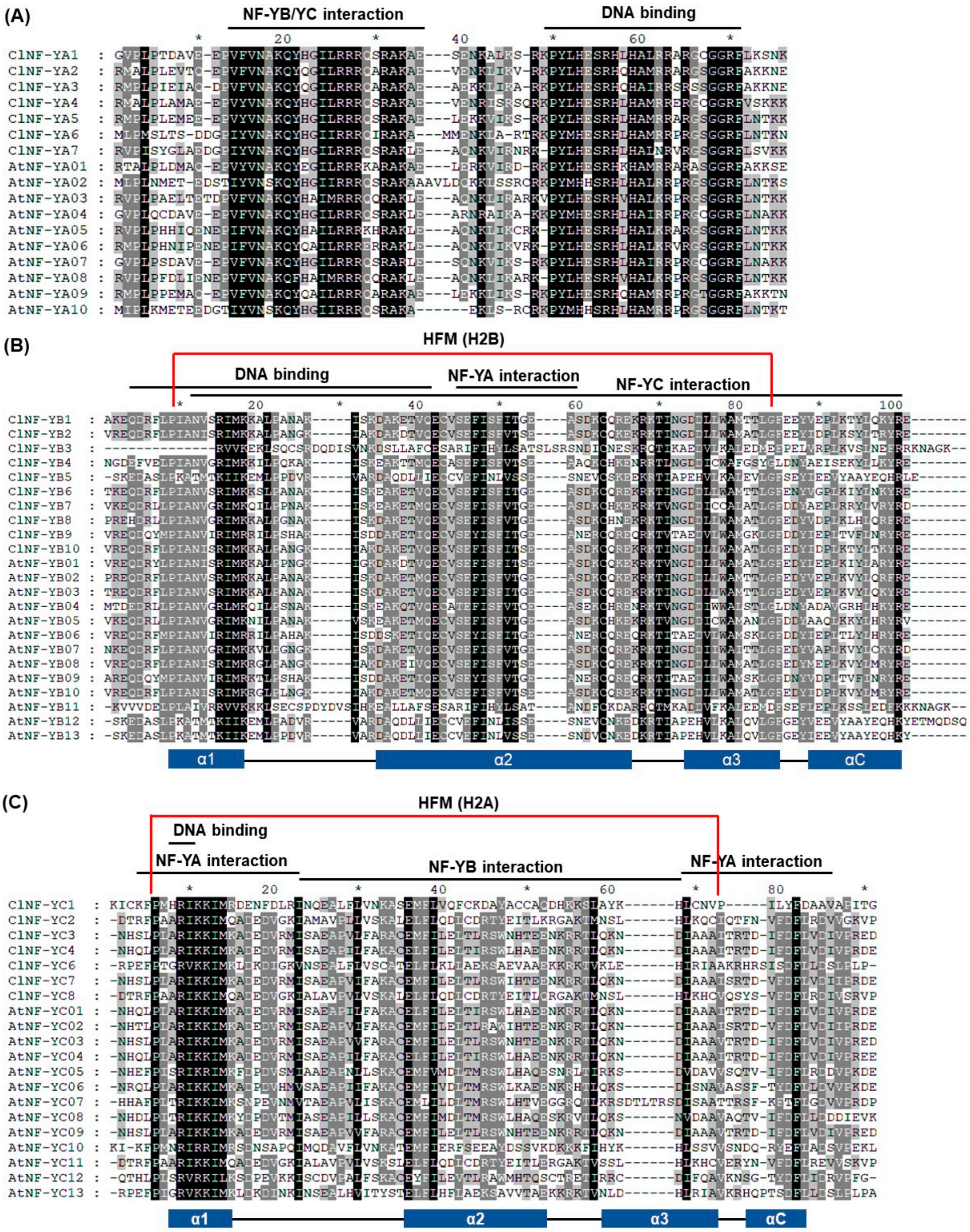

Figure S3. Sequence alignments of the conserved domains in watermelon ClNF-YAs, ClNF-YBs, and ClNF-YCs with Arabidopsis AtNF-Ys. (A) ClNF-YA subfamily; (B) ClNF-YB subfamily; (C) ClNF-YC subfamily. Multiple sequences alignment was constructed by DNAMAN software. The completely conserved amino acids are colored by black boxes and the relatively conserved amino acids (80%-100%) are colored by grey boxes. Regions responsible for subunit interaction and DNA binding are marked above the sequences and the structures of the conserved domains in ClNF-YBs and ClNF-YCs are indicated below the sequences.

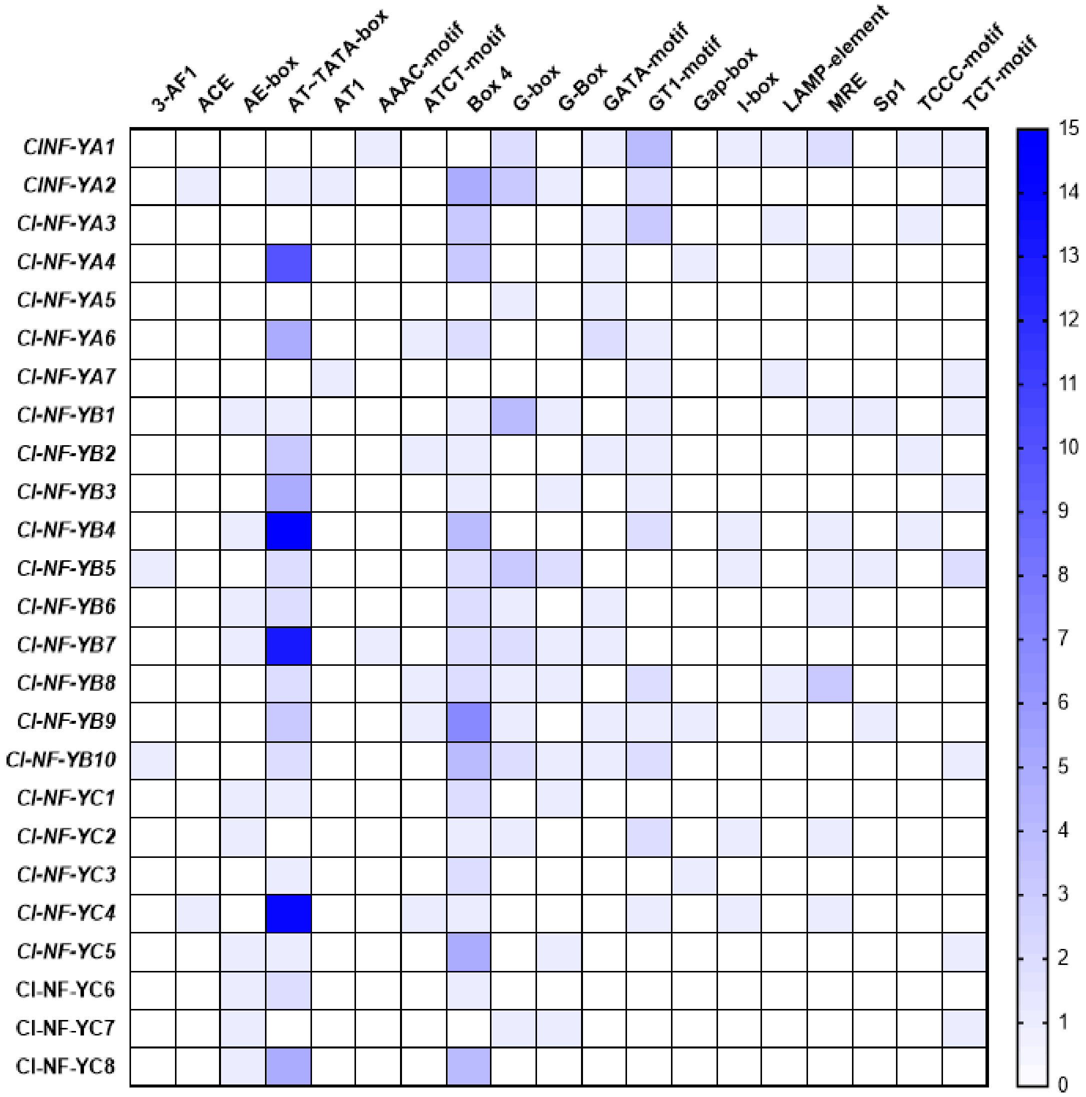

Figure S4. Light-responsive *cis*-elements present in promoters of the watermelon *ClNF-Y* genes.

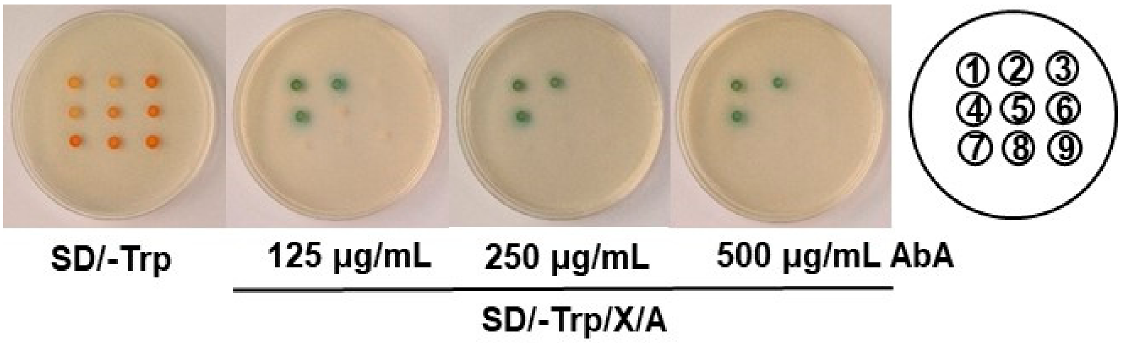

Figure S5. Autoactivation activity of ClNF-YCs in Y2H assays. Yeast cells were transformed with pGBKT7-ClNF-YCs and grown on SD/-Trp medium. Autoactivation activity was visualized by the appearance of blue color of the yeast cells after adding 40 μg/mL X-α-gal. Different concentrations of AbA were supplemented into SD/-Trp medium to inhibit putative autoactivation activity. Experiments were repeated three times with similar results.

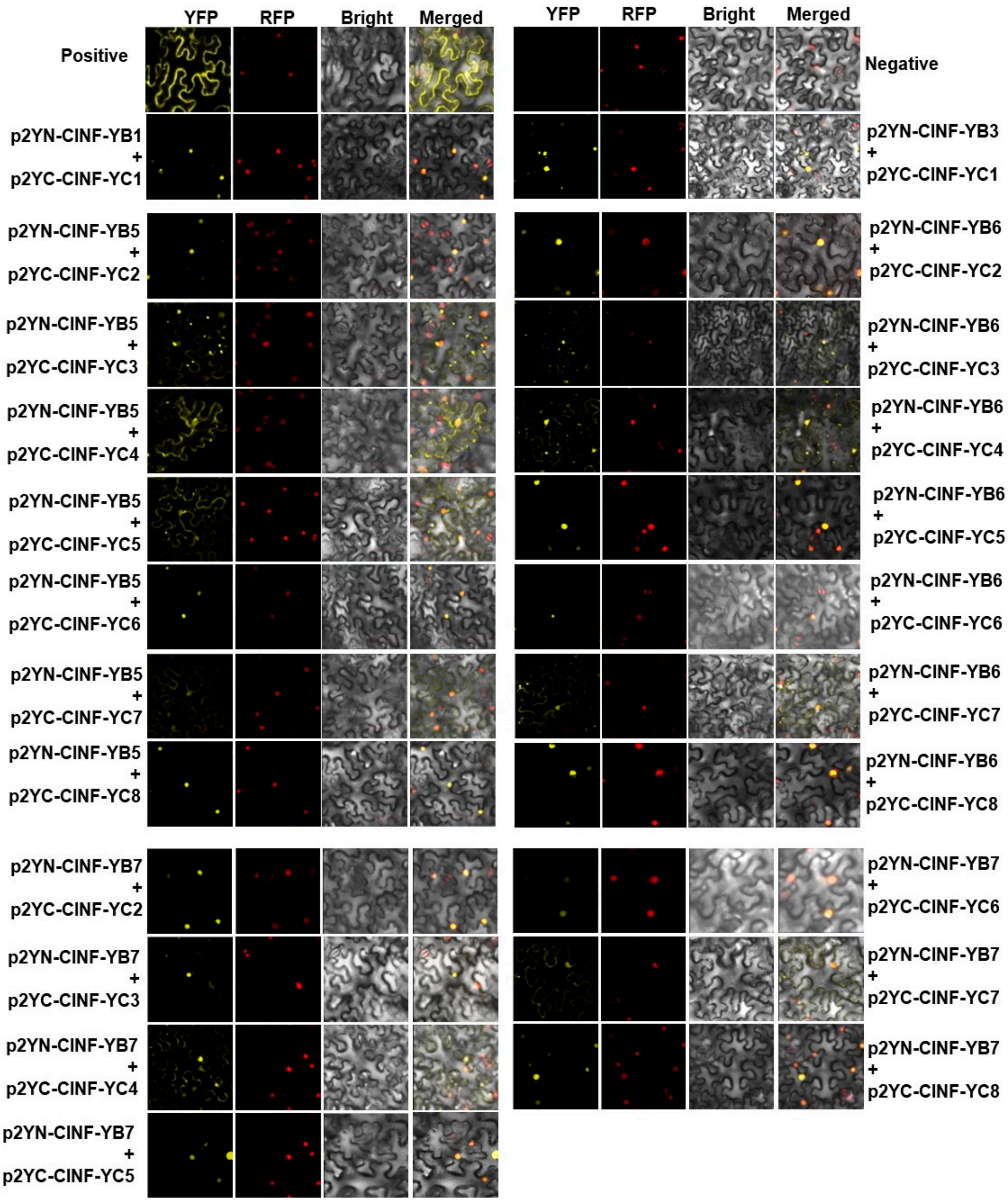

(*Continued*, see next page for legend)

(*Continued*)

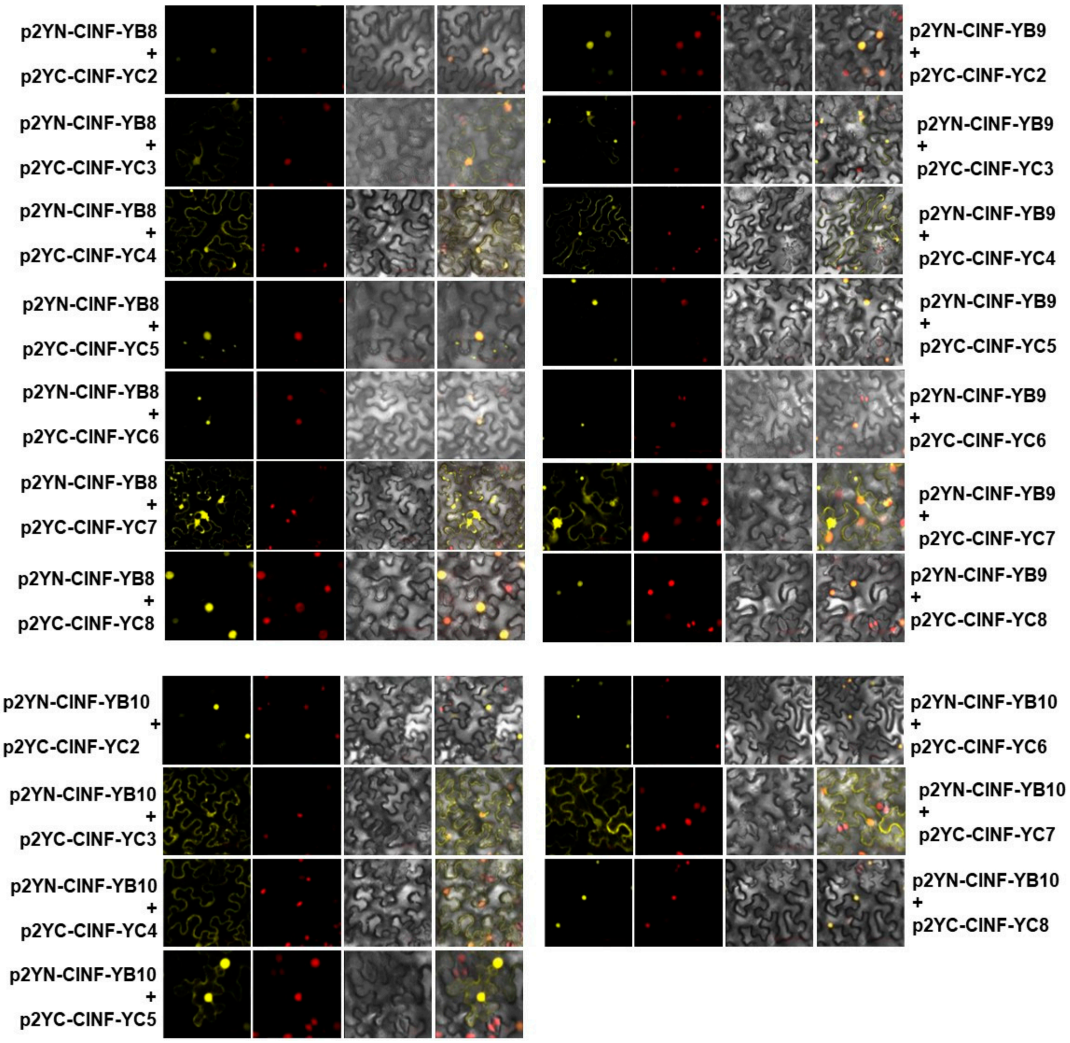

Figure S6. Interactions between ClNF-YBs and ClNF-YCs in BiFC assays. Experiments were performed three times with similar results.

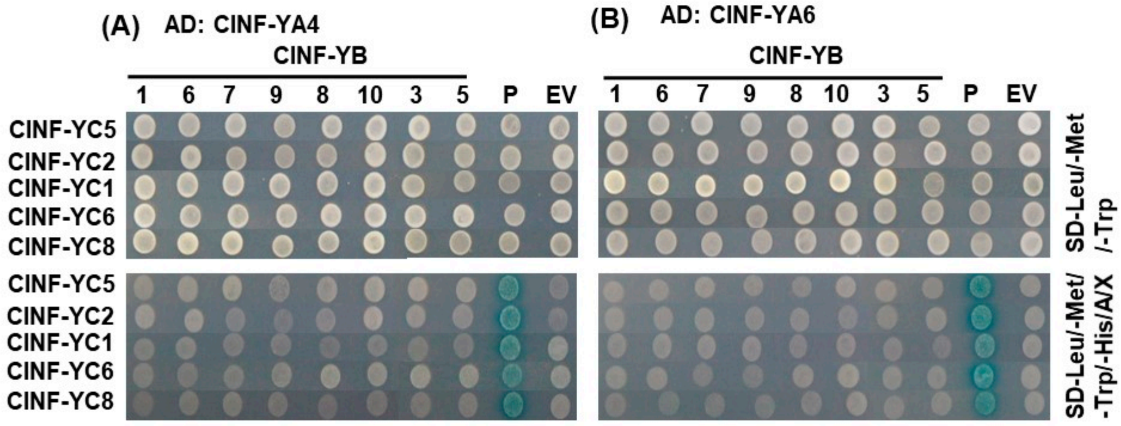

Figure S7. ClNF-YA4 and -YA6 did not assemble into ClNF-Y complexes with any of the ClNF-YB/-YC heterodimers in Y3H assays. ClNF-YA4 and ClNF-YA6 were cloned into pGADT7 to fuse with the GAL4 activation domain, while paired combinations of ClNF-YBs and ClNF-YCs were cloned into pBridge to fuse with the GAL4 DNA binding domain. Growth of yeasts co-transformed with pGADT7 and pBridge vectors were grown on SD-Leu/-Met/-Trp noninteraction selective medium or on SD-Leu/-Met/-Trp/-His/A/X interaction selective medium. Experiments were performed three times with similar results.

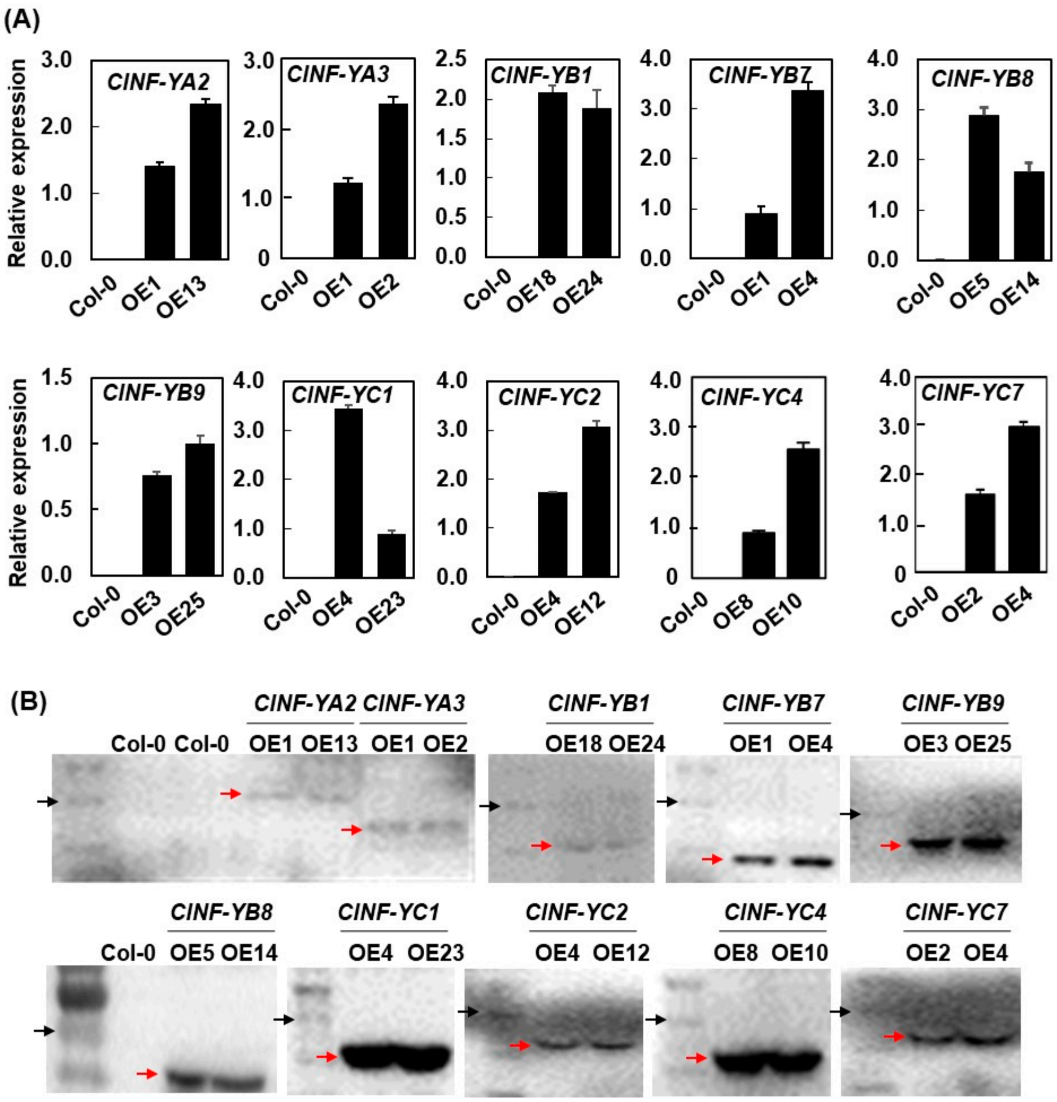

Figure S8. Characterization of the *ClNF-Y*-overexpressing transgenic Arabidopsis lines. (A) Transcript levels of the *ClNF-Y* genes in their corresponding transgenic Arabidopsis lines. Leaf samples were collected from four-week-old Arabidopsis plants, and qRT-PCR (B) analyses were performed with the Arabidopsis *AtActin* gene as an internal control. (B) Detection of the ClNF-Y-GFP fusion proteins in transgenic Arabidopsis lines by Western blotting using anti-GFP antibody. Black arrows indicate the molecular weight of 63 kDa, while the red arrows indicate the target fusion proteins. Experiments were repeated three times with similar results and the data presented are the means ± SE from three independent experiments.

Table S1. Ka/Ks ratio of the *ClNF-Y* gene pairs.

Gene pairs	Ka	Ks	Ka/Ks	Types of selection
ClNF-YA2-ClNF-YA3	0.2122	0.9165	0.2315	Purifying selection
ClNF-YA2-ClNF-YA5	0.4201	1.2898	0.3257	Purifying selection
ClNF-YB2-ClNF-YB10	0.1909	1.701	0.1122	Purifying selection

Table S2. Collinear gene pairs between watermelon (*Citrullus lanatus*) and *Arabidopsis thaliana*, rice (*Oryza sativa*), and cucumber (*Cucumis sativus*), and their Ka/Ks ratios.

ID	Genes	ID	Genes	Ka	Ks	Ka/Ks
Citrullus lanatus		***Arabidopsis thaliana***				
Cla97C01G025170	*ClNF-YA1*	AT1G30500	*AtNF-YA7*	Na	Na	Na
Cla97C01G025170	*ClNF-YA1*	AT2G34720	*AtNF-YA4*	0.3314	1.8808	0.1762
Cla97C02G049270	*ClNF-YA2*	AT3G20910	*AtNF-YA9*	0.3865	1.2602	0.3067
Cla97C02G049270	*ClNF-YA2*	AT5G12840	*AtNF-YA1*	0.3597	2.0487	0.1756
Cla97C03G063440	*ClNF-YA3*	AT3G20910	*AtNF-YA9*	0.3981	1.3213	0.3013
Cla97C03G063440	*ClNF-YA3*	AT5G12840	*AtNF-YA1*	0.3820	1.5179	0.2517
Cla97C10G201180	*ClNF-YA6*	AT5G06510	*AtNF-YA10*	0.3833	1.6131	0.2376
Cla97C02G039490	*ClNF-YB2*	AT2G37060	*AtNF-YB8*	0.2017	3.9198	0.0515
Cla97C02G048360	*ClNF-YB3*	AT2G27470	*AtNF-YB11*	Na	Na	Na
Cla97C03G067370	*ClNF-YB4*	AT1G09030	*AtNF-YB4*	0.3787	1.9730	0.1919
Cla97C06G124610	*ClNF-YB5*	AT5G08190	*AtNF-YB12,*	Na	Na	Na
Cla97C09G168740	*ClNF-YB7*	AT2G47810	*AtNF-YB5*	Na	Na	Na
Cla97C10G203800	*ClNF-YB10*	AT2G37060	*AtNF-YB8*	0.0944	1.6693	0.0566
Cla97C10G203800	*ClNF-YB10*	AT3G53340	*AtNF-YB10*	0.1578	1.8270	0.0864
Cla97C02G047110	*ClNF-YC1*	AT1G07980	*AtNF-YC10*	Na	Na	Na
Cla97C03G067230	*ClNF-YC3*	AT1G08970	*AtNF-YC9*	Na	Na	Na
Cla97C03G067230	*ClNF-YC3*	AT1G54830	*AtNF-YC3,*	Na	Na	Na
Cla97C06G127670	*ClNF-YC4*	AT3G48590	*AtNF-YC1*	Na	Na	Na
Cla97C06G127670	*ClNF-YC4*	AT5G63470	*AtNF-YC4*	0.0950	2.3127	0.0411
Citrullus lanatus		***Oryza sativa***				
Cla97C01G025170	*ClNF-YA1*	LOC_Os12g41880	*OsNF-YA9*	0.2284	Na	Na
Cla97C10G201180	*ClNF-YA6*	LOC_Os07g06470	*OsNF-YA5*	0.2892	Na	Na
Cla97C11G207940	*ClNF-YA7*	LOC_Os02g53620	*OsNF-YA11*	0.3244	2.3306	0.1392
Cla97C02G039490	*ClNF-YB2*	LOC_Os01g61810	*OsNF-YB2*	0.2157	Na	Na
Cla97C09G168740	*ClNF-YB7*	LOC_Os01g70890	*OsNF-YB6*	0.1924	Na	Na
Cla97C10G203800	*ClNF-YB10*	LOC_Os01g61810	*OsNF-YB2*	0.2101	Na	Na
Cla97C10G203800	*ClNF-YB10*	LOC_Os05g38820	*OsNF-YB3*	0.0636	Na	Na
Citrullus lanatus		***Cucumis sativus***				
Cla97C01G025170	*ClNF-YA1*	CsaV3_6G052320		0.0222	0.2111	0.1051
Cla97C02G049270	*ClNF-YA2*	CsaV3_6G039350		0.0260	0.1527	0.1700
Cla97C02G049270	*ClNF-YA2*	CsaV3_7G031020		0.5621	1.0997	0.5111
Cla97C03G063440	*ClNF-YA3*	CsaV3_1G042800		0.0250	0.2033	0.1230
Cla97C04G068610	*ClNF-YA4*	CsaV3_4G011610		0.1213	0.2783	0.4359
Cla97C09G166390	*ClNF-YA5*	CsaV3_7G031020		0.0391	0.1883	0.2077
Cla97C10G201180	*ClNF-YA6*	CsaV3_3G038100		0.0340	0.2110	0.1609
Cla97C02G035590	*ClNF-YB1*	CsaV3_6G009670		0.0342	0.2858	0.1198
Cla97C02G039490	*ClNF-YB2*	CsaV3_3G035490		0.1891	2.2902	0.0826

Cla97C02G039490	*ClNF-YB2*	CsaV3_5G006310	0.0563	0.3626	0.1553
Cla97C02G048360	*ClNF-YB3*	CsaV3_6G038390	0.0502	0.2052	0.2446
Cla97C03G067370	*ClNF-YB4*	CsaV3_1G038920	0.1801	0.3509	0.5133
Cla97C06G124610	*ClNF-YB5*	CsaV3_1G003510	0.0082	0.1965	0.0415
Cla97C07G136140	*ClNF-YB6*	CsaV3_4G004940	0.0368	0.4065	0.0906
Cla97C07G136140	*ClNF-YB6*	CsaV3_UNG179380	0.0368	0.4065	0.0906
Cla97C09G168740	*ClNF-YB7*	CsaV3_7G028980	0.0693	0.3517	0.1971
Cla97C10G188720	*ClNF-YB8*	CsaV3_3G004080	0.0228	0.3014	0.0758
Cla97C10G188900	*ClNF-YB9*	CsaV3_3G004250	0.0422	0.4781	0.0882
Cla97C10G203800	*ClNF-YB10*	CsaV3_3G035490	0.0124	0.0737	0.1690
Cla97C10G203800	*ClNF-YB10*	CsaV3_5G006310	0.2230	1.4689	0.1518
Cla97C02G047110	*ClNF-YC1*	CsaV3_6G021790	0.0788	0.1997	0.3949
Cla97C02G048620	*ClNF-YC2*	CsaV3_6G038670	0.0496	0.2539	0.1953
Cla97C03G067230	*ClNF-YC3*	CsaV3_1G039060	0.0067	0.1954	0.0341
Cla97C06G127670	*ClNF-YC4*	CsaV3_1G000520	0.0039	0.2948	0.0133
Cla97C07G135160	*ClNF-YC5*	CsaV3_4G006020	0.0260	0.5215	0.0499
Cla97C09G176240	*ClNF-YC7*	CsaV3_7G006030	0.0097	0.2484	0.0390
Cla97C11G218950	*ClNF-YC8*	CsaV3_5G029530	0.0264	0.2257	0.1168

Table S3. *Cis*-elements in promoters of the watermelon *ClNF-Y* genes

Elements	Functions	*ClNF-YAs*							*ClNF-YBs*										*ClNF-YCs*							
		1	2	3	4	5	6	7	1	2	3	4	5	6	7	8	9	10	1	2	3	4	5	6	7	8
3-AF1	light responsive												1					1								
**A-box**	**cis-acting regulatory element**			**1**																			**1**		**1**	**1**
**AAGAA-motif**	**development-related motifs**	**4**	**3**	**1**	**1**	**1**	**2**		**2**	**2**	**1**	**1**		**2**		**2**				**2**	**2**	**3**		**1**	**1**	
ABRE3a	stress and hormonal response		1						1																1	
**ABRE**	**abscisic acid responsiveness**		**5**						**4**		**1**		**3**		**2**	**2**	**1**	**4**					**1**	**1**	**2**	
**ABRE4**	**stress and hormonal response**		**1**						**1**																**1**	
**AC-II**	**vascular-specific expression**			**1**																						
**AACA**	**seed storage protein genes**					**1**				**1**		**1**														
ACE	light responsiveness		1																			1				
AE-box	part of a module for light response								1			1		1	1				1	1			1	1	1	1
AP-1	regulation of ribonucleotide reductase		1																						1	
**ARE**	**essential for anaerobic induction**	**4**	**1**	**2**		**1**	**3**	**2**		**5**		**2**	**1**	**1**	**5**	**1**		**1**	**3**	**6**	**1**		**2**		**2**	
TATA	general *cis*-element		1			2		1		2	1	1	1	1	2		3							1	2	
AT~TATA-box	light responsive		1		10		5		1	3	5	15	2	2	13	2	3	2	1		1	14	1	2		5
AT1 motif	light-responsive		1					1																		
AT-rich element	AT-rich DNA binding protein								1	1		1			1							1				
**AT-rich**	**elicitor-mediated activation**														**1**											
AAAC-motif	light responsive element	1													1											
ATCT-motif	light responsive						1			1						1	1					1				
**AuxRR-core**	**auxin responsive**							**1**		**1**						**1**										**1**
Box 4	light responsive		5	3	3		2		1	1	1	4	2	2	2	2	7	4	2	1	2	1	5	1		4
CAAT-box	common cis-acting element	22	41	27	27	14	35	33	30	23	28	37	43	21	24	38	32	24	25	17	24	26	30	48	26	33

CAT-box	related to meristem expression						1	1					2					1							1	
CARE	involved in viral DNA replication								1								1									
CCAAT-box	MYBHv1 binding site			1		1					1				1						1					
CCGTCC-motif	related to meristem specific activation			1																			1		1	1
CCGTCC-box	meristem specific activation			1																			1		1	1
CGTCA-motif	MeJA-responsive			1		1	1		1		3						3	1			2	1		1	1	1
CTAG-motif	rearrange the free energy						1			1	1											1				
DRE core	dehydration-responsive	1																2		1						
F-box	involved in abiotic stress																							3		
ERE	ethylene-responsive			1	3	1				1		1	1	3	2	1	3	1				2	4			1
G-Box	light responsive		1						1		1		2		1	1		1	1				1		1	
G-box	light responsive	2	3			1			4				3	1	2	1	1	2		1					1	
GC-motif	waterlogging stress pathways																							1		
GA-motif	light responsive									1					1									1		
GARE-motif	gibberellin-responsive						1	1	1																1	
GATA-motif	part of a light responsive element	1		1	1	1	2			1				1	1		1	1								
GCN4_motif	involved in endosperm expression						1						2		1	1		1								
GT1-motif	light responsive	4	2	3			1	1	1	1	1	2				2	1	2		2		1				
Gap-box	necessary for light responsiveness				1												1				1					
I-box	part of a light responsive element	1										1	1							1		1				
LAMP-element	light responsive	1		1				1								1	1									
LTR	low-temperature responsive	1				3	1				1											2				
MBS	involved in drought-inducibility	2				1	1	2			2		1	1		1			1		1				1	
MBSI	flavonoid biosynthetic genes																							1		
MRE	light responsive	2			1				1			1	1	1		3				1		1				

Myc	water and drought responsive	2			1	1					1		1				1								1	
**MYC**	**abiotic stress responses**	**2**	**1**	**1**	**3**		**5**	**3**	**2**	**4**	**1**	**4**	**3**			**3**	**4**	**1**	**1**		**5**	**5**	**3**	**9**	**3**	**6**
Myb-binding site	activation of lysozyme enhancer	1		2		1	2	1	1	1						1			1		1			1	1	1
Myb	stress-associated	2	1	1		2	1	2			2		1	1		2			2		2				1	
**MYB**	**development and stress response**	**5**	**1**	**4**		**2**	**3**	**2**	**2**	**2**	**1**	**1**	**4**	**1**	**1**	**6**	**1**	**2**	**2**	**2**	**5**	**2**	**1**	**3**	**1**	**3**
**MYB recognition**	**DNA-binding specificities**			**1**		**1**					**1**				**1**						**1**					
**MYB-like**	**sequence-specific DNA binding**	**3**	**1**	**2**			**1**	**1**		**1**	**1**		**1**			**4**	**1**	**2**	**1**	**1**		**1**		**1**		
MSA-like	cell cycle regulation																									
**O2-site**	**zein metabolism regulation**			**2**				**1**			**1**										**1**	**1**		**1**		
**P-box**	**gibberellin-responsive element**	**2**		**1**			**1**					**1**				**1**			**1**				**1**	**1**		
**RY-element**	**seed-specific regulation**				**1**											**1**										
Sp1	light responsive element								1				1				1									
TATA-box	core promoter element	15	38	12	52	39	20	22	9	26	36	61	50	33	54	20	39	16	24	27	25	56	35	29	10	68
**TATC-box**	**gibberellin-responsive**	**1**	**1**		**1**								**1**													
**TC-rich repeats**	**defense and stress responsive**		**1**			**1**		**1**			**1**			**1**										**1**		
**TCA**	**salicylic acid responsive**				**1**		**1**			**2**	**1**						**1**				**2**				**1**	
TCA-element	salicylic acid responsive					1	2				3	1	1		1	2		2	1	1		3			1	2
TCCC-motif	light responsive	1		1						1		1														
TCT-motif	part of a light responsive element	1	1					1	1		1		2					1					1		1	
**TGA-element**	**auxin-responsive**	**1**						**1**	**1**									**2**					**2**	**1**	**1**	
**TGACG-motif**	**MeJA-responsive**					**1**	**1**		**1**		**3**						**3**	**1**			**2**	**1**		**1**	**1**	**1**
Unnamed 1	CGTGG	1	3						3		1		5	1	1	3	2		1	1					1	
Unnamed_1	GAATTTAATTAA																						1			
Unamed__1	GGATTTTACAGT																		1							
Unnamed__2	AACCTAACCT	1				1						1		1		1			1							

Unnamed__4	CTCC	11	3	8	5	16	10	3	11	8	8	10	5	7	5	10	2	9	8	1	5	8	12	6	1	2
Unnamed__6	taTAAATATCT		1				1		1				1				1									2
Unnamed__8																									1	
Unnamed__10																									1	
Unnamed__12																									1	
Unnamed__14																									1	
**W box**	**hormone-mediated signaling**	**1**						**1**	**2**	**1**			**2**			**2**	**2**		**2**			**1**	**1**	**1**		
**WUN-motif**	**wound-responsive**		**3**			**1**			**1**				**1**	**2**						**1**				**1**	**1**	
**box S**	**pathogen-inducible**	**1**			**1**											**1**	**1**									
chs-CMA1a	light responsive		1											1								1			1	1
chs-CMA2a	light responsive												1	1					1		1					
**circadian**	**circadian control**														**1**											
**STRE**	**stress response**	**2**	**2**	**2**		**2**	**4**	**1**	**2**			**2**	**2**	**2**	**1**	**1**	**1**	**4**	**3**	**1**	**1**	**3**	**1**		**5**	**1**
**WRE3**	**stress responsive**	**1**						**1**						**1**		**2**	**1**									
**as-1**	**development-related motifs**					**1**	**1**		**1**		**3**						**3**	**1**			**2**	**1**		**1**	**1**	**1**
**Plant Ap-2-like**	**floret development**							**1**																		
re2f-1											1													1		
**Subtotal**	Excluding CAAT- and TATA-boxes	63	47	43	33	45	56	31	52	42	49	52	55	35	48	62	52	49	35	24	39	57	41	43	46	36
**TOTAL**		100	126	82	112	98	111	86	91	91	113	150	148	89	126	120	123	89	84	68	88	139	106	120	82	137

Note: Growth- and development-related *cis*-elements are labeled with red color; hormone-responsive *cis*-elements are indicated with blue color; abiotic and biotic stress-responsive *cis*-elements are shown in purple color.

Summary: Total *cis*-elements for *ClNF-Y* gene promoters: 2679; total *cis*-elements for *ClNF-Y* gene promoters excluding CAAT- and TATA-boxes: 1135; average of *cis*-elements for *ClNF-Y* gene promoters excluding CAAT- and TATA-boxes: 45.4

Table S4. Primers used in this study.

Primer names	Sequence (5'-3')	Size (bp)
Cloning		
ClNF-YA1-F	ATGGCCTCCTTATATAATGGTGA	603
ClNF-YA1-R	TCAGTTCTCTGACGAAGCAAG	
ClNF-YA2-F	ATGCAGTCGAAGTCTAAAAGTG	1026
ClNF-YA2-R	TCACTTGATGGCAAGACGCC	
ClNF-YA3-F	ATGCAGTCAAAGTCTGAAACC	810
ClNF-YA3-R	CTATTCTGAGGCCTTGTTTGT	
ClNF-YA4-F	ATGATGAGCCCTGAAATAGAT	615
ClNF-YA4-R	TCATATATATGCAGATAAATGTAG	
ClNF-YA5-F	ATGCAATCAAAACCAGAAAATG	1026
ClNF-YA5-R	TCAATTGATGGGGATGGCCCT	
ClNF-YA6-F	ATGGCACCACAAACTGGCTATTTG	1167
ClNF-YA6-R	TTACTTCATACTTGATATCTGA	
ClNF-YA7-F	ATGACAATGACTTTGTCTGATAA	903
ClNF-YA7-R	CTATGTTCCGTTTCCGCCAT	
ClNF-YB1-F	ATGGCGGATTCTGATAACGAATC	618
ClNF-YB1-R	CTACCGGTGTCCGGTGCC	
ClNF-YB2-F	ATGGCGGAGCCTCCCACCAGT	498
ClNF-YB2-R	TTAGAGAAGAGAGATTATTACTTG	
ClNF-YB3-F	ATGGGAGATCAGCATAATGG	405
ClNF-YB3-R	TTAAAATTGATTATTGTGTTCTTG	
ClNF-YB4-F	ATGAGCGGCCACAAACGAAAC	678
ClNF-YB4-R	TCACCAACCAGAGCTCTGTT	
ClNF-YB5-F	ATGGATGAAAACACAGGCATG	480
ClNF-YB5-R	TCATGCATCATGATTATTATTATTAG	
ClNF-YB6-F	ATGTTGCTGACCCCAAACAAGC	627
ClNF-YB6-R	TCAAAGCTGGCTACCATTCC	
ClNF-YB7-F	ATGGCTGACTCCGACAACGATTC	528
ClNF-YB7-R	CTACCTGGGCCGACCCGTA	
ClNF-YB8-F	ATGGCTGATGCTCCGGCGAGT	528
ClNF-YB8-R	TTAATGCATATTATTATTCGAGAG	
ClNF-YB9-F	ATGGAGAAAAAAGTAGGTGCC	486
ClNF-YB9-R	TCAGTTATCACTGGCGCCAAT	
ClNF-YB10-F	ATGACCAAAATTATCAAAGAG	411
ClNF-YB10-R	TTAGCTCTCTAAACTTTGCTCAG	
ClNF-YC1-F	ATGGATCAGCAAGGGCATGGC	783
ClNF-YC1-R	CTATTGATCAGGAGGGGTTTG	
ClNF-YC2-F	ATGGACACCAACAATCAAGCCC	663
ClNF-YC2-R	CTACCTCTGACCATCAAGAT	
ClNF-YC3-F	ATGAGGCAAGCGGGAGCAT	354
ClNF-YC3-R	TCAGCAAGGAATGGACATAGT	

ClNF-YC4-F	ATGGATCAATCAGAGCGTTCTC	801
ClNF-YC4-R	CTATGCATCACTTTGCTGCTGA	
ClNF-YC5-F	ATGCAAGCTGATGAAGATGTAG	792
ClNF-YC5-R	CTAACCTTCTTCGTCGTAATCT	
ClNF-YC6-F	ATGGCTTCATCCAAAAAGTC	603
ClNF-YC6-R	CTATAACAACCAATGAAAATAGA	
ClNF-YC7-F	ATGGAGGAAGAGGAAACC	423
ClNF-YC7-R	CTATGACTCGTTGGTCTCCG	
ClNF-YC8-F	ATGCAAGCAGATGAGGATGTTG	801
ClNF-YC8-R	TTACTCCTCTTCATCATAATCTTC	
OE vectors		
ClNF-YA1-GFP-F	CCCGAGCTCATGGCCTCCTTATATAATGGTGA	603
ClNF-YA1-GFP-R	CCCGGTACCGTTCTCTGACGAAGCAAGATCG	
ClNF-YA2-GFP-F	CCCGAGCTCATGCAGTCGAAGTCTAAAAGTG	1026
ClNF-YA2-GFP-R	CCCGGTACCCTTGATGGCAAGACGCCTCTG	
ClNF-YA3-GFP-F	CCCGAGCTCATGCAGTCAAAGTCTGAAACC	810
ClNF-YA3-GFP-R	CCCGGTACCTTCTGAGGCCTTGTTTGTCAAG	
ClNF-YA4-GFP-F	CCCGAGCTCATGATGAGCCCTGAAATAGAT	615
ClNF-YA4-GFP-R	CCCGGTACCTATATATGCAGATAAATGTAGTTG	
ClNF-YA5-GFP-F	CCCGAGCTCATGCAATCAAAACCAGAAAATG	1026
ClNF-YA5-GFP-R	CCCGGTACCATTGATGGGGATGGCCCTCTG	
ClNF-YA6-GFP-F	CCCGAGCTCATGGCACCACAAACTGGCTATTTG	1167
ClNF-YA6-GFP-R	CCCGGTACCCTTCATACTTGATATCTGATTGG	
ClNF-YA7-GFP-F	CCCGAGCTCATGACAATGACTTTGTCTGATAA	903
ClNF-YA7-GFP-R	CCCGGTACCTGTTCCGTTTCCGCCATGTAC	
ClNF-YB1-GFP-F	CCCGAGCTCATGGCGGATTCTGATAACGAATC	618
ClNF-YB1-GFP-R	CCCGGTACCCCGGTGTCCGGTGCCGGAACC	
ClNF-YB2-GFP-F	CCCGAGCTCATGGCGGAGCCTCCCACCAGT	498
ClNF-YB2-GFP-R	CCCGGTACCGAGAAGAGAGATTATTACTTG	
ClNF-YB3-GFP-F	CCCGAGCTCATGGGAGATCAGCATAATGG	405
ClNF-YB3-GFP-R	CCCGGTACCAAATTGATTATTGTGTTCTTG	
ClNF-YB4-GFP-F	AGCTTTCGCGAGCTCGGTACCATGAGCGGCCACAAAC GAAAC	678
ClNF-YB4-GFP-R	GCCCTTGCTCACCATGGTACCCCAACCAGAGCTCTGTT GAAG	
ClNF-YB5-GFP-F	AGCTTTCGCGAGCTCGGTACCATGGATGAAAACACAG GCATG	480
ClNF-YB5-GFP-R	GCCCTTGCTCACCATGGTACCTGCATCATGATTATTATT ATTAG	
ClNF-YB6-GFP-F	CCCGAGCTCATGTTGCTGACCCCAAACAAGC	627
ClNF-YB6-GFP-R	CCCGGTACCAAGCTGGCTACCATTCCCCGC	
ClNF-YB7-GFP-F	CCCGAGCTCATGGCTGACTCCGACAACGATTC	528
ClNF-YB7-GFP-R	CCCGGTACCCCTGGGCCGACCCGTAGTAG	
ClNF-YB8-GFP-F	CCCGAGCTCATGGCTGATGCTCCGGCGAGT	528

ClNF-YB8-GFP-R	CCCGGTACCATGCATATTATTATTCGAGAG	
ClNF-YB9-GFP-F	CCCGAGCTCATGGAGAAAAAGTAGGTGCC	486
ClNF-YB9-GFP-R	CCCGGTACCGTTATCACTGGCGCCAATTTC	
ClNF-YB10-GFP-F	CCCGAGCTCATGACCAAAATTATCAAAGAG	411
ClNF-YB10-GFP-R	CCCGGTACCGCTCTCTAAACTTTGCTCAG	
ClNF-YC1-GFP-F	AGCTTTCGCGAGCTCGGTACCATGGATCAGCAAGGGC ATGGC	783
ClNF-YC1-GFP-R	GCCCTTGCTCACCATGGTACC TTGATCAGGAGGGGTTTGATC	
ClNF-YC2-GFP-F	AGCTTTCGCGAGCTCGGTACCATGGACACCAACAATCA AGCCC	663
ClNF-YC2-GFP-R	GCCCTTGCTCACCATGGTACC CCTCTGACCATCAAGATTACC	
ClNF-YC3-GFP-F	CCCGAGCTCATGAGGCAAGCGGGAGCAT	354
ClNF-YC3-GFP-R	CCCGGTACCGCAAGGAATGGACATAG	
ClNF-YC4-GFP-F	CCCGAGCTCATGGATCAATCAGAGCGTTCTC	801
ClNF-YC4-GFP-R	CCCGGTACCTGCATCACTTTGCTGCTGAGTTTG	
ClNF-YC5-GFP-F	CCCGAGCTCATGCAAGCTGATGAAGATGTAG	792
ClNF-YC5-GFP-R	CCCGGTACCACCTTCTTCGTCGTAATCTTCC	
ClNF-YC6-GFP-F	CCCGAGCTCATGGCTTCATCCAAAAAGTC	603
ClNF-YC6-GFP-R	CCCGGTACCTAACAACCAATGAAAATAGAC	
ClNF-YC7-GFP-F	AGCTTTCGCGAGCTCGGTACCATGGAGGAAGAGGAAA CC	423
ClNF-YC7-GFP-R	GCCCTTGCTCACCATGGTACCTGACTCGTTGGTCTCCG C	
ClNF-YC8-GFP-F	CCCGAGCTCATGCAAGCAGATGAGGATGTTG	801
ClNF-YC8-GFP-R	CCCGGTACCCTCCTCTTCATCATAATCTTC	
BiFC assays		
ClNF-YB1-P2YN-1F	CCCTTAATTAACATGGCGGATTCTGATAACGAATC	618
ClNF-YB1-P2YN-1R	GGGACTAGTCCGGTGTCCGGTGCCG	
ClNF-YB2-P2YN-1F	CCCTTAATTAACATGGCGGAGCCTCCCA	498
ClNF-YB2-P2YN-1R	GGGACTAGTGAGAAGAGAGATTATTACTTGAGTGTTTA	
ClNF-YB3-P2YN-1F	CCCTTAATTAACATGGGAGATCAGCATAATGGG	405
ClNF-YB3-P2YN-1R	GGGACTAGTAAATTGATTATTGTGTTCTTGTTCATC	
ClNF-YB4-P2YN-1F	CCCTTAATTAACATGAGCGGCCACAAACGAAACT	678
ClNF-YB4-P2YN-1R	GGGACTAGTCCAACCAGAGCTCTGTTGAAGATG	
ClNF-YB5-P2YN-1F	CCCTTAATTAACATGGATGAAAACACAGGCA	480
ClNF-YB5-P2YN-1R	GGGACTAGTTGCATCATGATTATTATTATTAGTATTA	
ClNF-YB6-P2YN-1F	CCCTTAATTAACATGTTGCTGACCCCAAACAAGC	627
ClNF-YB6-P2YN-1R	GGGACTAGTAAGCTGGCTACCATTCCCCG	
ClNF-YB7-P2YN-1F	CCCTTAATTAACATGGCTGACTCCGACAACGATC	528
ClNF-YB7-P2YN-1R	GGGACTAGTCCTGGGCCGACCCGTAGTAGTA	
ClNF-YB8-P2YN-F	CCCTTAATTAACATGGCTGATGCTCCGGC	528
ClNF-YB8-P2YN-R	GGGACTAGTATGCATATTATTATTCGAGAGTGTCTGT	

ClNF-YB9-P2YN-F	CCCTTAATTAACATGGAGAAAAAAGTAGGTGCCGG	486
ClNF-YB9-P2YN-R	GGGACTAGTGTTATCACTGGCGCCAATTTCAT	
ClNF-YB10-P2YN-F	CCCTTAATTAACATGACCAAAATTATCAAAGAGATGTTACC	411
ClNF-YB10-P2YN-R	GGGACTAGTGCTCTCTAAACTTTGCTCAGGCTCC	
ClNF-YC1-P2YC-F	CCCTTAATTAACATGGATCAGCAAGGGCATGG	783
ClNF-YC1-P2YC-R	GGGACTAGTTTGATCAGGAGGGGTTTGATCTTG	
ClNF-YC2-P2YC-F	CCCTTAATTAACATGGACACCAACAATCAAGCC	663
ClNF-YC2-P2YC-R	GGGACTAGTCCTCTGACCATCAAGATTACCC	
ClNF-YC3-P2YC-F	CCCTTAATTAACATGAGGCAAGCGGGAGCA	354
ClNF-YC3-P2YC-R	GGGACTAGTGCAAGGAATGGACATAGTTTCAGTT	
ClNF-YC4-P2YC-F	CCCTTAATTAACATGGATCAATCAGAGCGTTCTCAG	801
ClNF-YC4-P2YC-R	GGGACTAGTTGCATCACTTTGCTGCTGAGTTT	
ClNF-YC5-P2YC-F	CCCTTAATTAACATGCAAGCTGATGAAGATGTAGGG	792
ClNF-YC5-P2YC-R	GGGACTAGTACCTTCTTCGTCGTAATCTTCCTCG	
ClNF-YC6-P2YC-F	CCCTTAATTAACATGGCTTCATCCAAAAAGTCAA	603
ClNF-YC6-P2YC-R	GGGACTAGTTAACAACCAATGAAAATAGACAGCAG	
ClNF-YC7-P2YC-F	CCCTTAATTAACATGGAGGAAGAGGAAACCGG	423
ClNF-YC7-P2YC-R	GGGACTAGTTGACTCGTTGGTCTCCGCC	
ClNF-YC8-P2YC-F	CCCTTAATTAACATGCAAGCAGATGAGGATGTTG	801
ClNF-YC8-P2YC-R	GGGACTAGTCTCCTCTTCATCATAATCTTCCTCTTC	
Y3H assays		
ClNF-YA1-AD-F	GCCATGGAGGCCAGTGAATTCATGGCCTCCTTATATAATGGTGA	603
ClNF-YA1-AD-R	ATGCCCACCCGGGTGGAATTCTCAGTTCTCTGACGAAGCAAGA	
ClNF-YA2-AD-F	GCCATGGAGGCCAGTGAATTCATGCAGTCGAAGTCTAAAAGTG	1026
ClNF-YA2-AD-R	ATGCCCACCCGGGTGGAATTCTCACTTGATGGCAAGACGCC	
ClNF-YA3-AD-F	GCCATGGAGGCCAGTGAATTCATGCAGTCAAAGTCTGAAACC	810
ClNF-YA3-AD-R	ATGCCCACCCGGGTGGAATTCCTATTCTGAGGCCTTGTTTGT	
ClNF-YA4-AD-F	GCCATGGAGGCCAGTGAATTCATGATGAGCCCTGAAATAGATT	615
ClNF-YA4-AD-R	ATGCCCACCCGGGTGGAATTCTCATATATATGCAGATAAATGTAG	
ClNF-YA5-AD-F	CCCGAATTCATGCAATCAAAACCAGAAAATGT	1026
ClNF-YA5-AD-R	CCCGAATTCTCAATTGATGGGGATGGCCCT	
ClNF-YA6-AD-F	CCCGAATTCATGGCACCACAAACTGGCTA	1167
ClNF-YA6-AD-R	CCCGAATTCTTACTTCATACTTGATATCTGA	
ClNF-YA7-AD-F	GCCATGGAGGCCAGTGAATTCATGACAATGACTTTGTCTGATA	903

ClNF-YA7-AD-R	ATGCCCACCCGGGTGGAATTCCTATGTTCCGTTTCCGCCAT	
ClNF-YC1-BD-F	CCCGAATTCATGGATCAGCAAGGGCATGG	783
ClNF-YC1-BD-R	CCCGAATTCCTATTGATCAGGAGGGGTTTG	
ClNF-YC2-BD-F	ATGGCCATGGAGGCCGAATTCATGGACACCAACAATCAAGCC	663
ClNF-YC2-BD-R	TCGACGGATCCCCGGGAATTCCTACCTCTGACCATCAAGAT	
ClNF-YC3-BD-F	CCCGAATTCATGAGGCAAGCGGGAGCAT	354
ClNF-YC3-BD-R	CCCGAATTCTCAGCAAGGAATGGACATAGT	
ClNF-YC4-BD-F	CCCGAATTCATGGATCAATCAGAGCGTTCTC	801
ClNF-YC4-BD-R	CCCGAATTCCTATGCATCACTTTGCTGCTGA	
ClNF-YC5-BD-F	CCCGAATTCATGCAAGCTGATGAAGATGTAG	792
ClNF-YC5-BD-R	CCCGAATTCCTAACCTTCTTCGTCGTAATCT	
ClNF-YC6-BD-F	CCCGAATTCATGGCTTCATCCAAAAAGTCAA	603
ClNF-YC6-BD-R	CCCGAATTCCTATAACAACCAATGAAAATAGA	
ClNF-YC7-BD-F	ATGGCCATGGAGGCCGAATTCATGGAGGAAGAGGAAACCGG	423
ClNF-YC7-BD-R	TCGACGGATCCCCGGGAATTCCTATGACTCGTTGGTCTCCG	
ClNF-YC8-BD-F	ATGGCCATGGAGGCCGAATTCATGCAAGCAGATGAGGATGTTG	801
ClNF-YC8-BD-R	TCGACGGATCCCCGGGAATTCTTACTCCTCTTCATCATAATCTTC	
ClNF-YB1-pBridge-F	CCCGAATTCATGGCGGATTCTGATAACGAA	618
ClNF-YB1-pBridge-R	CCCGAATTCCTACCGGTGTCCGGTGCC	
ClNF-YB2-pBridge-F	TTGACTGTATCGCCGGAATTCATGGCGGAGCCTCCCACCAGT	498
ClNF-YB2-pBridge-R	TCGACGGATCCCCGGGAATTCTTAGAGAAGAGAGATTATTACTTG	
ClNF-YB3-pBridge-F	CCCGAATTCATGGGAGATCAGCATAATGG	405
ClNF-YB3-pBridge-R	CCCGAATTCTTAAAATTGATTATTGTGTTCTTG	
ClNF-YB4-pBridge-F	TTGACTGTATCGCCGGAATTCATGAGCGGCCACAAACGAAAC	678
ClNF-YB4-pBridge-R	TCGACGGATCCCCGGGAATTCTCACCAACCAGAGCTCTGTTG	
ClNF-YB5-pBridge-F	CCCGAATTCATGGATGAAAACACAGGCATGTC	480
ClNF-YB5-pBridge-R	CCCGAATTCTCATGCATCATGATTATTATTATTAG	
ClNF-YB6-pBridge-F	TTGACTGTATCGCCGGAATTCATGTTGCTGACCCCAAACAAGC	627
ClNF-YB6-pBridge-R	TCGACGGATCCCCGGGAATTCTCAAAGCTGGCTACCATTCCCC	
ClNF-YB7-pBridge-F	CCCGAATTCATGGCTGACTCCGACAACGAT	528

ClNF-YB7-pBridge-R	CCCGAATTCCTACCTGGGCCGACCCGTA	
ClNF-YB8-pBridge-F	CCCGAATTCATGGCTGATGCTCCGGCGA	528
ClNF-YB8-pBridge-R	CCCGAATTCTTAATGCATATTATTATTCGAGAG	
ClNF-YB9-pBridge-F	TTGACTGTATCGCCGGAATTCATGGAGAAAAAGTAG GTGCC	486
ClNF-YB9-pBridge-R	TCGACGGATCCCCGGGAATTCTCAGTTATCACTGGCGC CAATT	
ClNF-YB10--pBridge-F	CCCGAATTCATGACCAAAATTATCAAAGAGATG	411
ClNF-YB10--pBridge-R	CCCGAATTCTTAGCTCTCTAAACTTTGCTCAG	
ClNF-YC1-pBridge-F	CCCGCGGCCGCAATGGATCAGCAAGGGCATGG	783
ClNF-YC1-pBridge-R	CCCAGATCTCTATTGATCAGGAGGGGTTTG	
ClNF-YC2-pBridge-F	CCCGCGGCCGCATGGACACCAACAATCAAGCC	663
ClNF-YC2-pBridge-R	CCCAGATCTCTACCTCTGACCATCAAGAT	
ClNF-YC3-pBridge-F	CCCGCGGCCGCAATGAGGCAAGCGGGAGCAT	354
ClNF-YC3-pBridge-R	CCCAGATCTTCAGCAAGGAATGGACATAGT	
ClNF-YC4-pBridge-F	CCCGCGGCCGCAATGGATCAATCAGAGCGTTCTC	801
ClNF-YC4-pBridge-R	CCCAGATCTCTATGCATCACTTTGCTGCTGA	
ClNF-YC5-pBridge-F	CCCGCGGCCGCAATGCAAGCTGATGAAGATGTAG	792
ClNF-YC5-pBridge-R	CCCAGATCTCTAACCTTCTTCGTCGTAATCT	
ClNF-YC6-pBridge-F	CCCGCGGCCGCAATGGCTTCATCCAAAAAGTCAA	603
ClNF-YC6-pBridge-R	CCCAGATCTCTATAACAACCAATGAAAATAGA	
ClNF-YC7-pBridge-F	CCCGCGGCCGCATGGAGGAAGAGGAAACCGG	423
ClNF-YC7-pBridge-R	CCCAGATCTCTATGACTCGTTGGTCTCCG	
ClNF-YC8-pBridge-F	CCCGCGGCCGCATGCAAGCAGATGAGGATGTTG	801
ClNF-YC8-pBridge-R	CCCAGATCTTTACTCCTCTTCATCATAATCTTC	
RT-qPCR		
ClNF-YA1-RT-F	GGTGGACGGTTTCTCAA	110
ClNF-YA1-RT-R	GCAAGATCGCTTCTATCAGA	
ClNF-YA2-RT-F	GCAGTAGCCAATTCCATAA	105
ClNF-YA2-RT-R	CACTTCGCTGCTGTCCC	
ClNF-YA3-RT-F	TGGAGGACGTTTTGCTA	112
ClNF-YA3-RT-R	GCTTCCGTTCTGCTGTTC	
ClNF-YA4-RT-F	GATGATGATGATGTGGAAGG	100
ClNF-YA4-RT-R	CAGATAAATGTAGTTGGGAAG	
ClNF-YA5-RT-F	ACTCAAAATATGCAGATAACAC	144
ClNF-YA5-RT-R	TGGAGCCCCATTCACTT	
ClNF-YA6-RT-F	GATAGTCCCCGCGTTTG	130
ClNF-YA6-RT-R	GGATGATTCCTTTGTTTCTC	
ClNF-YA7-RT-F	GACTGCAACTTGCTCCG	115
ClNF-YA7-RT-R	ATACTGCTCCATTCTCCC	
ClNF-YB1-RT-F	CGGCAGTGGGACAAATA	149
ClNF-YB1-RT-R	CACCAGCACCATAAACG	
ClNF-YB2-RT-F	CGACGTTGGGATTTGAG	148

ClNF-YB2-RT-R	TGCTGGGAATTTTGACC	
ClNF-YB3-RT-F	ATGGCTTAGATAACTATGCTG	109
ClNF-YB3-RT-R	TGTAGTTGTTGAGTAATTTTGC	
ClNF-YB4-RT-F	TCAGGGGAACCAGAAGA	131
ClNF-YB4-RT-R	GAAGATGAGCCGCCATA	
ClNF-YB5-RT-F	TGATGATATTTGCTGTGCT	119
ClNF-YB5-RT-R	ATCCCTTATTTGTTGAGC	
ClNF-YB6-RT-F	GCATTTCAAATAGGGCACG	105
ClNF-YB6-RT-R	AAGCTGGCTACCATTCC	
ClNF-YB7-RT-F	GGCGGTGGGGAATTTGG	112
ClNF-YB7-RT-R	ACCTGGGCCGACCCGTAGT	
ClNF-YB8-RT-F	CTGCTAAAGGTGGTGATGC	139
ClNF-YB8-RT-R	AGTACTTGAGAACTCGCATAA	
ClNF-YB9-RT-F	GCAAGAATGCCGGAAAG	117
ClNF-YB9-RT-R	TCGGCTTCATCATTATCC	
ClNF-YB10-RT-F	AAGGTGGGAAGTGGAGTA	115
ClNF-YB10-RT-R	GCTTCGGTGCAGTGTTA	
ClNF-YC1-RT-F	GGTAAGCCTGTGATGGAC	100
ClNF-YC1-RT-R	GATCAGGAGGGGTTTGA	
ClNF-YC2-RT-F	GGAGACCGGCAATGGAT	103
ClNF-YC2-RT-R	CCACTCGCGTAAGAACC	
ClNF-YC3-RT-F	GGCCACTGATGTTTTCG	101
ClNF-YC3-RT-R	AGCAAGGAATGGACATAGTTT	
ClNF-YC4-RT-F	ATGGGGAAGCAATTAGAT	104
ClNF-YC4-RT-R	TGCTGCTGAGGTTGAGTAT	
ClNF-YC5-RT-F	CGAATATCCTGGTTGGTC	102
ClNF-YC5-RT-R	TCGTAATCTTCCTCGTCAT	
ClNF-YC6-RT-F	ATCGGATCAAGAAAATCAT	132
ClNF-YC6-RT-R	GAGCACAGCACGCATAT	
ClNF-YC7-RT-F	ACTCGCACTGTCGTTGA	105
ClNF-YC7-RT-R	CTATGACTCGTTGGTCTCC	
ClNF-YC8-RT-F	TACCACCACCACCAGCAG	101
ClNF-YC8-RT-R	AGGCCAGGCAGGGAATT	
ClGAPDH-F	ATGGGCAAAGTTAAGATCGGCATCA	91
ClGAPDH-R	CCAATTCGATATCATCACTCTGC	
AtPR1-RT-F	TCGTCTTTGTAGCTCTTGTAGGTG	120
AtPR1-RT-R	TAGATTCTCGTAATCTCAGCTCT	
AtPR5-RT-F	ATGGCA AATATCTCCAGTATTCACA	125
AtPR5-RT-R	ATGTCGGGGCAAGCCGCGTTGAGG	
AtActin-F	GGCGATGAAGCTCAATCCAAACG	110
AtActin-R	GGTCACGACCAGCAAGATCAAGACG	
